# Genomic insights into the virulence and salt tolerance of *Staphylococcus equorum*

**DOI:** 10.1038/s41598-017-05918-5

**Published:** 2017-07-14

**Authors:** Do-Won Jeong, Sojeong Heo, Sangryeol Ryu, Jochen Blom, Jong-Hoon Lee

**Affiliations:** 1 Department of Food and Nutrition, Dongduk Women’s University, Seoul, 02748 Republic of Korea; 2 Department of Food Science and Biotechnology, Kyonggi University, Suwon, 16227 Republic of Korea; 3 Department of Food and Animal Biotechnology, Department of Agricultural Biotechnology, and Center for Food and Bioconvergence, Seoul National University, Seoul, 08826 Republic of Korea; 4 Bioinformatics and System Biology, Justus-Liebig-University Giessen, 35392 Giessen, Germany

## Abstract

To shed light on the genetic background behind the virulence and salt tolerance of *Staphylococcus equorum*, we performed comparative genome analysis of six *S*. *equorum* strains. Data on four previously published genome sequences were obtained from the NCBI database, while those on strain KM1031 displaying resistance to multiple antibiotics and strain C2014 causing haemolysis were determined in this study. Examination of the pan-genome of five of the six *S*. *equorum* strains showed that the conserved core genome retained the genes for general physiological processes and survival of the species. In this comparative genomic analysis, the factors that distinguish the strains from each other, including acquired genomic factors in mobile elements, were identified. Additionally, the high salt tolerance of strains enabling growth at a NaCl concentration of 25% (w/v) was attributed to the genes encoding potassium voltage-gated channels. Among the six strains, KS1039 does not possess any of the functional virulence determinants expressed in the other strains.

## Introduction

*Staphylococcus equorum*, initially isolated from a healthy horse^1^, is a common component of the microbiota in the fermented foods of Europe, including fermented meat products^2–4^ and smear-ripened and semi-hard cheeses^5, 6^. This species has been reported to produce low-molecular-weight aromatic compounds, such as esters, amino acids, aldehydes and free fatty acids, in food fermentation^7, 8^. Recently, *S*. *equorum* has also been identified as the dominant species of jeotgal, a high-salt-fermented seafood of Korea^9^.

Coagulase-negative staphylococci (CNS) including *S*. *equorum* are generally known as benign bacteria, in contrast to the coagulase-producing *Staphylococcus aureus*. No staphylococcal food poisoning via fermented foods has been attributed to *S*. *equorum*, and no evidence of its pathogenicity has been reported. However, the emergence of strains suspected of being involved in bovine mastitis^10^, showing high prevalence of acquired phenotypes including antibiotic resistance and haemolysis^11–13^, has necessitated safety assessments of this species. In this context, safety assessments of starter candidates for traditional Spanish dry-cured sausages, dairy products, jeotgal and fermented meat products have been performed^12–15^.

Analyses of the genome sequences of three *S*. *equorum* strains have led to reports that the strains do not possess any of the virulence factors found in *S*. *aureus* ^10, 16, 17^. However, the genomic data are insufficient to identify the features of strains from different niches and those suspected of being pathogenic. In the current study, we thus extended the analyses to the genomes of two *S*. *equorum* strains showing resistance to multiple antibiotics or causing haemolysis. We also performed a comparative genomic analysis of *S*. *equorum* strains together with the four previously reported genomes to define the scale and scope of the pan-genome and the core genes and clarify the genetic background behind such antibiotic resistance and haemolysis. This study introduced intraspecific comprehensive comparative genome analysis to shed light on the genetic background behind the phenotypic characteristics.

## Methods

### Bacterial strains and culture conditions

Two *S*. *equorum* strains, KM1031 and C2014 isolated and characterized in our previous studies^9, 13^, were subjected to genomic analysis. Strain KM1031 isolated from Myeolchi-jeotgal, a Korean high-salt-fermented anchovy, exhibited resistance to chloramphenicol, erythromycin, lincomycin and penicillin G; strain C2014 from Saeu-jeotgal, a Korean high-salt-fermented tiny sea shrimp, induced haemolysis on sheep blood-supplemented tryptic soy agar (TSA; Difco, Detroit, MI, USA) plates. For experimental proof of the genomic analysis results, strains KS1039 from Saeu-jeotgal^16^, Mu2 from French smear-ripened cheese^17^, and UMC-CNS-924 from bovine mastitis^10^ were used. *S*. *equorum* subsp. *linens* separated from *S*. *equorum* in 2003^6^ was reunified into a species in 2013^18^. *S*. *equorum* strains were cultured in tryptic soy broth (TSB; Difco) at 30 °C for 24 h to maintain their traits^13^. *Escherichia coli* BL21(DE3) was used as the cloning host and was incubated in Luria–Bertani (LB) medium (Difco) at 37 °C for 12 h.

### Genome sequencing

Genomic DNA was isolated and purified using a Wizard Genomic DNA Purification Kit (Promega, Madison, WI, USA). The concentration and purity of the extracted DNA were determined using a Quanti-iT PicoGreen dsDNA Assay Kit (Invitrogen, Carlsbad, CA, USA), and the contamination of DNA was checked by sequencing the 16 S rRNA gene using an ABI 3730 DNA sequencing machine (Applied Biosystems, Foster City, CA, USA). Whole-genome sequencing was performed using a combination of the Illumina Miseq system (150 bp paired end) and the PacBio Single-Molecule Real-Time (SMRT) sequencing system (20 kbp) at ChunLab, Inc. (Seoul, South Korea). Four and six contigs were generated from a hybrid assembly of reads from the Illumina system (4,663,200 reads and >341.43 coverage for KM1031 strain; 3,873,510 reads and >287.08 coverage for C2014 strain) and PacBio system (75,778 reads and >400.53 coverage for KM1031 strain; 71,229 reads and >389.10 coverage for C2014 strain) for *S*. *equorum* strains KM1031 and C2014, respectively. The reads were assembled using CLC Genomics Workbench ver. 7.5.1 (CLC Bio, Aarhus, Denmark) and CodonCode Aligner (CodonCode Co., Centerville, MA, USA). Gene prediction was performed using Glimmer 3^19^, followed by annotation through a search against the Clusters of Orthologous Groups (COG)^20^ and SEED databases^21^.

### Comparative genomics

For comparative genomic analysis within the species *S*. *equorum*, the genome sequence data of strains KS1039 (GenBank accession: CP013114.1), Mu2 (CAJL00000000.1), UMC-CNS-924 (AVBD00000000.1) and G8HB1 (LAKE00000000.1) published before July 2016 were obtained from the NCBI database (http://ncbi.nlm.nih.gov/genomes). The genome sequence data of 15 strains published after September 2016 were also used for additional comparative genomic analysis^22^. The average nucleotide identity (ANI), which provides a robust measurement of genetic distance among bacterial genomes, among the conserved genes of the genomes was used for comparative analysis^23^. To determine the rearrangements in each genome, a progressive alignment algorithm implemented in MAUVE^24^ was used. The Efficient Database framework for comparative Genome Analyses using BLAST score Ratios (EDGAR) was used for core genome, pan-genome and singleton analyses^25^; the genome of strain KS1039 was used as a reference genome for Venn diagram construction. Comparative analyses at the protein level were performed by an all-against-all comparison of the annotated genomes. The algorithm used was BLASTP and were normalized according to the best score^26^. The score ratio value, which shows the quality of the hit, was calculated by dividing the scores of further hits by the best hit^27^. Two genes were considered orthologous when revealing a bidirectional best BLAST hit with a single score ratio value threshold of at least 32% for orthology estimation.

### Haemolysis activity test

TSA supplemented with 5% sheep blood (v/v) (BBL Microbiology Systems, Sparks, MD, USA) was used for β- and δ-haemolytic activity tests. β-Haemolytic activity was determined by cold shock at 4 °C for 24 h after incubation at 30 °C for 24 h; δ-haemolytic activity was determined by cross-streaking the test strains perpendicularly to *S*. *aureus* RN4220 at 30 °C for 24 h. The same method was implemented in the selection of *S*. *equorum* strains having β- and δ-haemolytic activities^13^. Experiments were conducted three times, on separate days.

### Growth monitoring in the presence of antibiotics and NaCl

*S*. *equorum* strains cultured in TSB were normalized to 0.5 turbidity at OD_600_ and then diluted 1:100 in TSB supplemented with antibiotics to confirm the function of annotated antibiotic resistance genes. The antibiotics chloramphenicol, ciprofloxacin, erythromycin, lincomycin, methicillin, penicillin G and tetracycline were purchased from Sigma (St. Louis, MO, USA) and employed at concentrations of 30, 5, 15, 30, 8, 6 and 30 μg/ml, respectively, based on previous research results^11, 13^ and the guidelines of methicillin for *S*. *aureus* set out by the Clinical and Laboratory Standards Institute^28^. The salt tolerance of *S*. *equorum* strains was determined by examining their growth in TSB supplemented with NaCl at concentrations of 15% (w/v), 20% and 25%. To determine the salt tolerance of *E*. *coli* transformants, NaCl was employed at a final concentration of 3% or 6% in LB broth. Cell growth was monitored by measuring OD_600_ using a Varioskan Flash (Thermo Scientific, Waltham, MA, USA).

### Cloning of the potassium voltage-gated channel genes

The potassium voltage-gated channel genes of strains KM1031 and C2014 were amplified with specific primer sets (Supplementary Table S1) designed from their genome sequences. PCR amplifications were performed using a T-3000 thermocycler (Biometra, Göttingen, Germany) and the PCR mixture consisted of the template DNA, 0.5 μM of each primer, 1.25 units of Inclone *Taq* polymerase (Inclone Biotech, Daejeon, South Korea), 10 mM dNTPs and 2 mM MgCl_2_. Samples were preheated for 5 min at 95 °C and then amplified using 30 cycles of 1 min at 95 °C, 30 s at 55 °C and 1 min at 72 °C. The amplified PCR products were cloned into pGEM-T Easy vector (Promega, Madison, WI, USA) under the control of the T7 promoter. The successful integration of the cloned fragments was confirmed by sequencing using GenoTech (Daejeon, South Korea) with the T7 primer set.

### Nucleotide sequence accession numbers

The complete genome sequences and annotation data of *S*. *equorum* strains KM1031 and C2014 have been deposited in GenBank under the accession numbers CP013980–CP013983 and CP013714–CP013719, respectively.

## Results and Discussion

### Genome summary and general features

The general features of the genomes of the six *S*. *equorum* strains including strains KM1031 and C2014 are summarized in Table 1. The genome (2,792,213 bp) of *S*. *equorum* KM1031 consists of a single circular DNA chromosome of 2,693,398 bp with G + C content of 33.1% and three plasmids. The genome contains 2,642 predicted open reading frames (ORFs), 60 tRNAs and 22 rRNAs. The genome (2,930,519 bp) of *S*. *equorum* C2014 consists of a single circular DNA chromosome of 2,753,539 bp with G + C content of 32.9% and five plasmids. The genome contains 2,846 predicted ORFs, 59 tRNAs and 22 rRNAs. In total, 2,295 and 2,431 protein-coding sequences (CDSs) were predicted from the genome sequences of strains KM1031 and C2014 containing 2,642 and 2,846 ORFs, respectively, with 86.9% and 85.4% being assigned a COG functional classification.

**Table 1:** General genomic and specific phenotypic features of six *Staphylococcus equorum* strains.

Feature	KM1031	C2014	KS1039	Mu2	UMC-CNS-924	G8HB1
Size (bp)	2,792,213	2,930,519	2,822,193	2,927,171	2,700,865	2,799,869
Chromosome size (bp)	2,693,398	2,753,539	2,822,193	ND	ND	ND
G + C content (%)	33.05	32.85	33.07	32.80	32.96	33.08
No. of plasmids	3^a^	5^b^	0	ND	4	ND
Open reading frames	2,642	2,846	2,681	2,745	2,499	2,621
CDSs assigned by COG	2,295	2,431	2,363	2,469	2,332	2,408
CDSs assigned by SEED	1,990	2,009	2,009	2,045	1,940	1,994
No. of rRNAs	22	22	22	4	24	8
No. of tRNAs	60	59	61	55	57	54
Contigs	4	6	1	30	39	22
Scaffolds	0	0	0	30	39	22
Origin	Myeolchi-jeotgal	Saeu-jeotgal	Saeu-jeotgal	French smear-ripened cheese	Milk from Holstein cow	Human gall bladder
Specific phenotypic features^c^						
Antibiotic resistance	Chl^r^, Ery^r^, Lin^r^, Pen^r^	−	−	−	Lin^r^, Tet^r^	NT
Haemolysis	−	β-Haemolysis	−	−	δ-Haemolysis	NT
Growth on 25% NaCl	−	+	+	−	–	NT

^a^Plasmids in the strain KM1031: pKM1031-1, 45.9 kb; pKM1031-2, 50.2 kb; and pSELNU3, 2.6 kb. ^b^Plasmids in the strain C2014: pC2014-1, 80.3 kb; pC2014-2, 64.4 kb; pC2014-3, 13.0 kb; pC2014-4, 7.3 kb; and pC2014-5, 12.0 kb. ^c^Phenotypic characteristics were reconfirmed in this study, except for strain G8HB1. Abbreviations: Chl, chloramphenicol; Ery, erythromycin; Lin, lincomycin; Pen, penicillin G; Tet, tetracycline; ND, not determined by the authors; NT, not tested in this study; +, positive; −, negative.

The average genome sequence length of the six strains is 2,828,805 bp. *S*. *equorum* UMC-CNS-924 exhibits the smallest genome (2,700,865 bp), while strain C2014 possesses the largest one (2,930,519 bp). All *S*. *equorum* strains display average G + C content of 33%.

To facilitate a coherent comparative analysis, we performed consistent ORF prediction for the six *S*. *equorum* (complete and incomplete) genome sequences. In this way, comparable numbers of genes were obtained for each genome, with an average of 2,672 ORFs per genome (Table 1). Notably, (BLAST-based) functional in silico prediction could be performed for 89.2% of the identified ORFs, while the remaining 10.8% not assigned a COG functional classification were predicted to encode hypothetical proteins.

**Figure 1:**
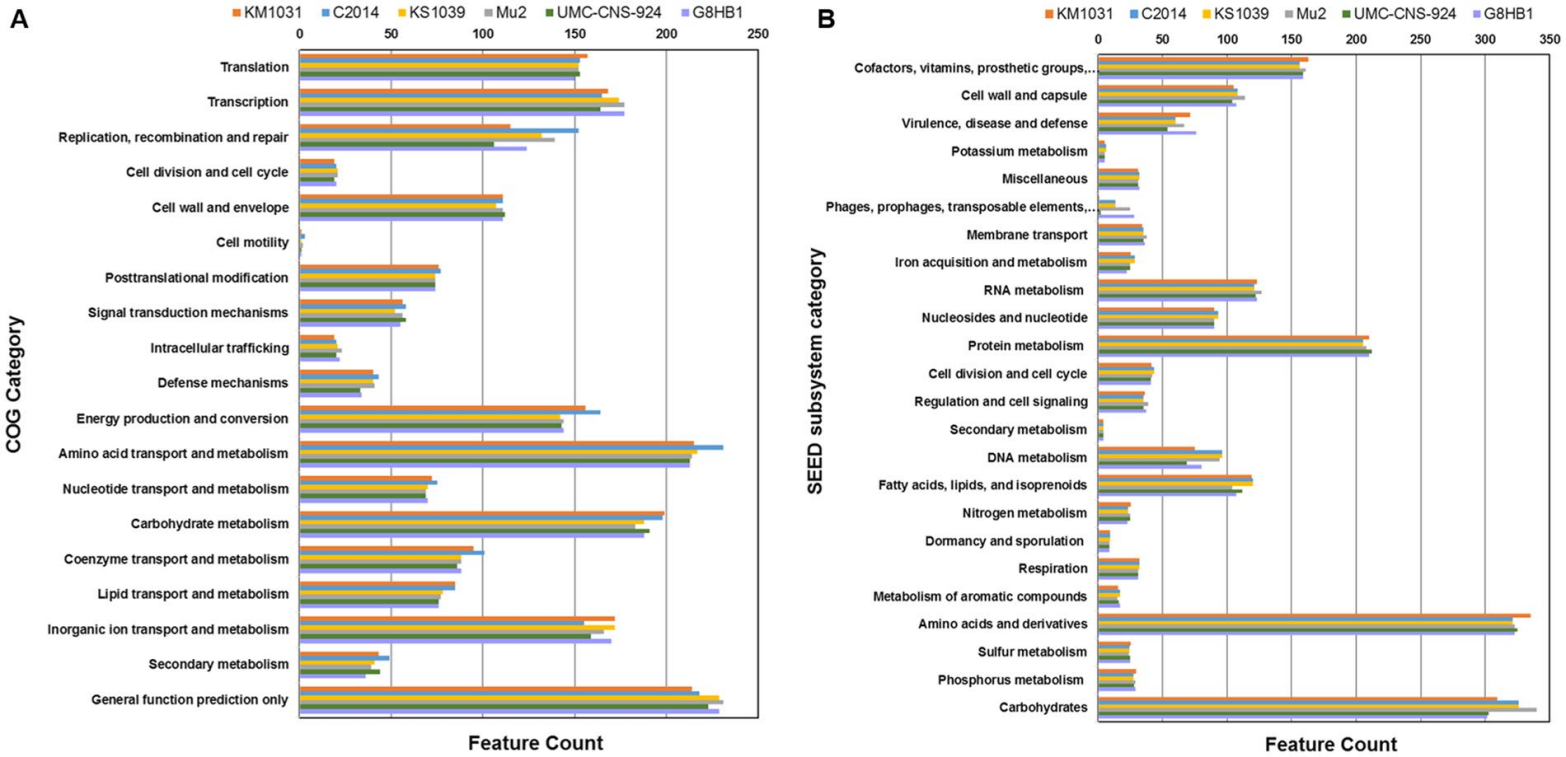
Comparison of functional categories in six *S*. *equorum* genomes based on COG (**A**) and SEED (**B**). Genome sequences of six strains KM1031, C2014, KS1039, Mu2, UMC-CNS-924 and G8HB1 were uploaded to the COG and SEED viewer servers independently. Functional roles of annotated genes were assigned and grouped in subsystem feature categories. Coloured bars indicate the number of genes assigned to each category.

Analysis using the SEED subsystem categorization and COG functional categorization predicted the existence of an average of 1,998 CDSs and 2,383 CDSs per genome, respectively (Table 1). Based on the SEED subsystem, over 321 CDSs accounting for 15.8–16.8% of the *S*. *equorum* genomes were allocated to the genes for amino acid biosynthesis and utilization (Fig. 1). The next most abundant subsystem category is related to carbohydrate utilization (15.5–16.7%), followed by protein metabolism. Major COG subsystems are related to amino acid transport and metabolism, as well as carbohydrate transport and metabolism. Both analyses enabled coherent conclusions to be drawn regarding the subsystem category of *S*. *equorum*.

### Comparative analysis of *S*. *equorum* genomes

Whole-genome comparison of the six *S*. *equorum* strains showed that the genomes are highly homologous in terms of functional category (Fig. 1). MAUVE alignment of the six genomes allowed the identification of approximately 8–10 locally collinear blocks (LCBs), regions without rearrangement of the homologous backbone sequence (Supplementary Fig. S1). However, the LCBs are interspaced by specific DNA stretches of various lengths. MAUVE analysis showed an overall collinear relationship across *S*. *equorum* strains KM1031, C2014 and KS1039, which were isolated from different jeotgal samples^9, 13^. When the genome of strain KS1039 was established as the standard, a large-scale chromosomal reorganization by a single recombination event was found to have occurred in the genome of strain G8HB1, which resulted in the inversion of a genomic region; in addition, the results indicated that the genomes of strains Mu2 and UMC-CNS-924 might have been generated by complex rearrangements. The collinear relationship and complex rearrangement found in the six *S*. *equorum* genome structures can be explained by their differences in isolation source and geographic location. However, the contigs in genomic data for strains Mu2, UMC-CNS-924 and G8HB1 might have distorted the MAUVE analysis results.

**Figure 2:**
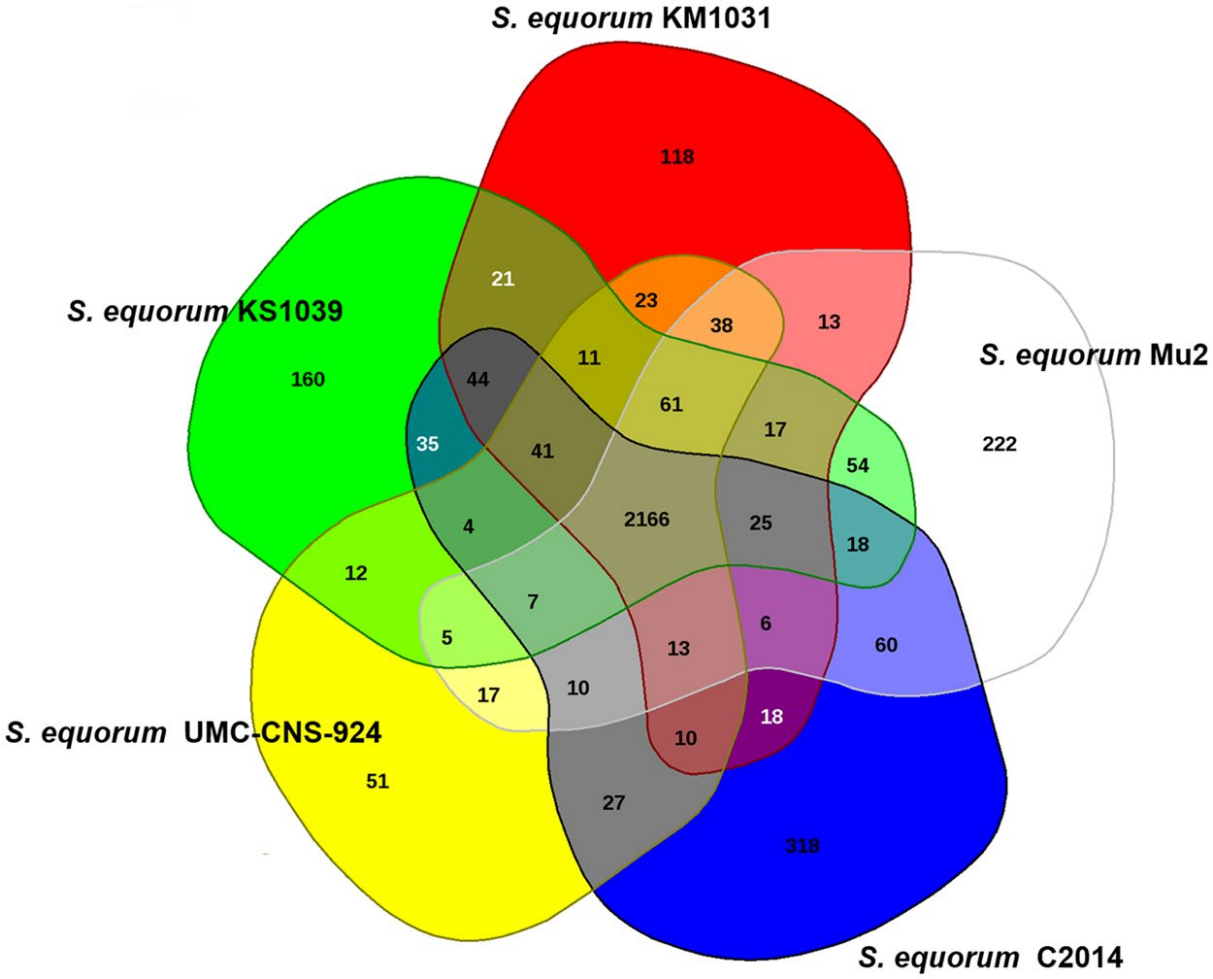
Venn diagram of five *S*. *equorum* genomes. The Venn diagram shows the pan-genome of strains KM1031, C2014, KS1039, Mu2 and UMC-CNS-924 generated using EDGAR. Overlapping regions represent common CDSs shared between the *S*. *equorum* genomes. The numbers outside the overlapping regions indicate the numbers of CDSs in each genome without homologs in the other sequenced *S*. *equorum* genomes.

The gene pools shared by the genomes of the five *S*. *equorum* strains KM1031, C2014, KS1039, Mu2 and UMC-CNS-924 are depicted in a Venn diagram (Fig. 2). These five strains share 2,166 CDSs in their core genome, corresponding to approximately 76.1–86.7% of their ORFs. Many of the CDSs in the core genome are assigned via COG annotation to functions relating to metabolism and the transport of amino acids and carbohydrates. The genome of strain UMC-CNS-924 has the smallest proportion (2.0%) of unique CDSs that are absent from the four other *S*. *equorum* genomes. In contrast, the proportions of unique CDSs in the genomes of strains KM1031, C2014, KS1039 and Mu2 are 4.5%, 10.9%, 6.0% and 8.1%, respectively. The majority of singleton-specific genes are associated with hypothetical proteins (Supplementary Table S2). Meanwhile, functional singletons in the genomes of strains KM1031, C2014, KS1039, UMC-CNS-924 and Mu2 are allocated to transporter, transposase, CRISPR-associated protein, tetracycline resistance and phage-related genes, respectively.

### Insights into virulence

The well-known food pathogen *S*. *aureus* produces several virulence factors: a clumping factor to protect against phagocytosis, an extracellular adhesion protein for adhesion, an enterotoxin, a toxic shock syndrome toxin, and cytotoxins such as α- and β-haemolysins for tissue invasion^29^. However, genomic analysis revealed that the six *S*. *equorum* strains do not possess any of the virulence determinants for adhesion, enterotoxins and pathogenicity islands that are found in *S*. *aureus*. Nonetheless, strain C2014 induced haemolysis on sheep blood-supplemented agar and strain KM1031 displayed resistance to chloramphenicol, erythromycin, lincomycin and penicillin G^13^; therefore, we focused on genetic analysis to explain the differences in phenotype among the *S*. *equorum* strains.

#### Haemolysis

The α- and β-haemolysins are prevailing toxins of *S*. *aureus*, but their homologs were not identified in any of the six *S*. *equorum* genomes. However, three CDSs annotated as haemolysin, haemolysin III and haemolysin activation protein genes were identified in all of the genomes of five *S*. *equorum* strains, excluding strain KS1039 (Table 2). Strain KS1039 does not possess a predicted haemolysin gene. These three CDSs also exist in all of 15 *S*. *equorum* strains from cheeses (Supplementary Table S3).

**Table 2:** Potential virulence determinants identified in six *S*. *equorum* genomes.

Virulence factor	Gene locus					
	KM1031	C2014	KS1039	Mu2	UMC-CNS-924	G8HB1
Haemolysis-related						
Haemolysin	AWC34_RS03405	AVJ22_RS03205		SEQMU2_RS08595	SEQU_RS25035	UF72_RS01895
Haemolysin III	AWC34_RS09060	AVJ22_RS09420	SE1039_RS09485	SEQMU2_RS01345	SEQU_RS26575	UF72_RS08525
Haemolysin activation protein	AWC34_RS11995	AVJ22_RS12370	SE1039_RS12450	SEQMU2_RS04245	SEQU_RS16015	UF72_RS08300
					SEQU_RS16020	
					SEQU_RS16025	
Haemolysin family calcium-binding region		AVJ22_RS14095^*^				
Antibiotic resistance						
*Efflux pump*						
Chloramphenicol resistance protein DHA1	AWC34_RS10365	AVJ22_RS10770	SE1039_RS10790	SEQMU2_RS02670	SEQU_RS15060	UF72_RS09825
Lincomycin resistance protein LmrB	AWC34_RS12700	AVJ22_RS13165		SEQMU2_RS05490	SEQU_RS20895	UF72_RS07545
Quinolone resistance protein NorB	AWC34_RS00175	AVJ22_RS00115	SE1039_RS00130	SEQMU2_RS05750	SEQU_RS20615	UF72_RS07265
Quinolone resistance protein NorB	AWC34_RS10900	AVJ22_RS13260	SE1039_RS11310	SEQMU2_RS03165	SEQU_RS23900	UF72_RS10355
Multidrug resistance protein SepA	AWC34_RS09050	AVJ22_RS09410	SE1039_RS09475	SEQMU2_RS01335	SEQU_RS26585	UF72_RS08515
Multidrug resistance protein SMR	AWC34_RS00690	AVJ22_RS00690	SE1039_RS00770	SEQMU2_RS06295	SEQU_RS23055	UF72_RS06755
Antibiotic ABC transporter ATP-binding protein	AWC34_RS11115				SEQU_RS24115	UF72_RS10570
Tetracycline resistance MFS efflux pump					SEQU_RS26615^*^	
*Enzymatic inactivation*						
Methicillin resistance protein	AWC34_RS05640	AVJ22_RS05905	SE1039_RS06065	SEQMU2_RS11115	SEQU_RS17120	UF72_RS04430
Methicillin resistance protein	AWC34_RS05645	AVJ22_RS05910	SE1039_RS06070	SEQMU2_RS11120	SEQU_RS17125	UF72_RS04435
Methicillin resistance protein FemA	AWC34_RS11375	AVJ22_RS11680	SE1039_RS11790	SEQMU2_RS03635	SEQU_RS17305	UF72_RS10830
β-Lactamase	AWC34_RS13020^*^	AVJ22_RS12330		SEQMU2_RS04220	SEQU_RS22365	UF72_RS12370
Lincomycin resistance protein LnuA	AWC34_RS13300^*^				SEQU_RS26665^*^	
*Other*						
Antibiotic biosynthesis monooxygenase	AWC34_RS01805					

*Genes located in a plasmid.

The deduced amino acid sequence of annotated haemolysin gene (AVJ22-RS03205) from strain C2014 has 98% identity with the TlyC amino acid sequence of *Streptococcus equi*. TlyC was described as a haemolysin in *Brachyspira hyodysenteriae* and was considered to be a virulence factor contributing to the disease caused by this spirochete because *tlyC*-expressing *E*. *coli* displayed haemolytic activity *in vitro* ^30^. Meanwhile, Carvalho *et al*.^31^ concluded that TlyC of *Leptospira* does not exert a direct haemolytic effect but may contribute to *Leptospira* binding to the extracellular matrix during host infection. In addition, Turner and Helmann^29^ reported that YhdP (TlyC) in *Bacillus subtilis* is a multidrug efflux protein and its amino acid sequence has 67% similarity with that of a membrane protein in *Bacillus isronensis*. These results suggest that the haemolysin homolog may not contribute to haemolysin activity directly but may instead be a membrane protein or enhance haemolytic activity.

The integral membrane protein gene *hlyIII* was identified from *Bacillus cereus* and *Vibrio vulnificus*; its involvement in haemolysis was verified by its expression in a nonhaemolytic *E*. *coli* strain^30, 32^. However, its homolog found in *Bacteroides fragilis* was not linked to haemolytic activity^33^. Among the *S*. *equorum* strains tested for haemolytic activity, only strain C2014 exhibited β-haemolytic activity, despite all strains having the haemolysin III gene, which means that this gene is not an independent determinant of β-haemolysis.

All of the six *S*. *equorum* strains possess a gene encoding a putative haemolysin activation protein composed of 43 amino acids. However, only strain UMC-CNS-924 has three genes encoding three distinct haemolysin activation proteins under the control of a promoter (Table 2) and exhibited δ-haemolysis (Supplementary Fig. S2B). The three putative haemolysin activation proteins presented ≤45.5% amino acid sequence identities with each other and ≤37.2% sequence identity with that of strain C2014 (Supplementary Table S4). *Staphylococcus lugdunensis* was reported to secrete three 43-amino-acid peptides with synergistic haemolytic activity, phenotypically similar to the δ-haemolysin of *S*. *aureus*, and their genes are located in an operon^30^. The identification of δ-haemolysin activity only in strain UMC-CNS-924 may be attributable to these three genes, while their homologs found in the other five strains may contribute to other functions.

Among the strains tested for haemolytic activity, only strain C2014 exhibited β-haemolytic activity (Supplementary Fig. S2A). Comparative genomic analysis revealed an additional CDS only found in strain C2014, a putative haemolysin family calcium-binding region gene (AVJ22_RS14095) (Table 2) and a unique gene harboured in the plasmid named pC2014-5 (12.0 kb). The product of this gene was reported to induce stabilization of the β-sheet structure of haemolysin and be enhanced by Ca^2+^ binding^34^. Therefore, we assumed that the expression of the gene AVJ22_RS14095 may enhance the haemolytic activity of other gene products that cannot induce haemolysis on their own. Based on the SEED subsystem, the genes AVJ22_RS03205 and AVJ22_RS09420 in strain C2014 were classified as encoding magnesium/cobalt efflux protein and the membrane protein haemolysin III, respectively. Thus, we cautiously suggest that the gene AVJ22_RS09420 might be a determinant of the haemolysis activity, with the support of AVJ22_RS14095, in strain C2014.

#### Acquired antibiotic resistance

Based on the functional categories as determined by the SEED subsystem, an average of 65.5% of the annotated CDSs in the virulence, disease and defence category for the six *S*. *equorum* strains are predicted to be genes for resistance to antibiotics and toxic compounds (Fig. 1B). Meanwhile, the genes for resistance to antibiotics and toxic compounds in strains KM1031 and G8HB1 constitute 71.8% and 73.7% of the CDSs in this category, respectively.

**Figure 3:**
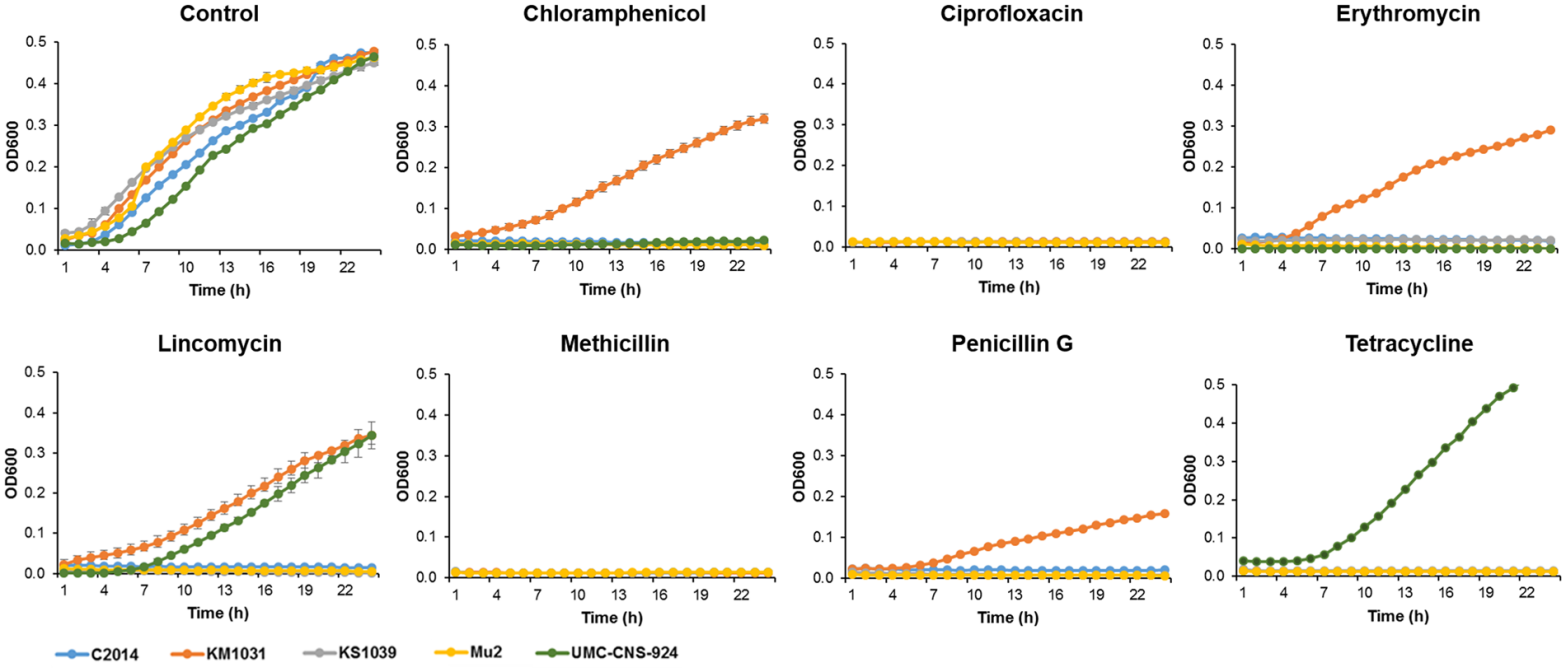
Growth of five *S*. *equorum* strains in the presence of antibiotics.

Putative efflux pump genes for chloramphenicol, lincomycin, quinolone and multiple drugs were identified across the six *S*. *equorum* chromosomes as well as those of 15 *S*. *equorum* strains from cheeses (Table 2, Supplementary Table S3). KM1039 is the only strain that does not harbour the putative lincomycin-resistance gene *lmrB*. Conversely, the phenotype of lincomycin resistance was exhibited in strains KM1031 and UMC-CNS-924 (Fig. 3). The lincomycin-resistant *S*. *equorum* KM1031 harbours a plasmid encoding the *lnuA* gene, which it can transfer to Gram-positive recipients^11^. Strain UMC-CNS-924 also harbours the *lnuA*-encoding plasmid. The lincomycin resistance of both strains might thus have been acquired via horizontal transfer of the resistant plasmid. The six commonly identified putative efflux pump genes including the *lmrB* homolog may thus not function in lincomycin resistance.

Resistance to ciprofloxacin, a kind of quinolone, was not identified in any of the test strains. In our previous antibiotic resistance test of 126 *S*. *equorum* strains, two strains showed independent ciprofloxacin and ofloxacin resistance and both strains also exhibited multidrug resistance^14^. Their quinolone resistance might be an acquired trait and the chromosomal quinolone resistance homologs may be weakly related to the phenotypic quinolone resistance of *S*. *equorum*.

Among the test strains, only the strain KM1031 exhibited resistance to chloramphenicol as well as erythromycin. Comparative genomic analysis has highlighted the putative antibiotic ABC transporter ATP-binding protein gene (AWC34_RS11115) and the antibiotic biosynthesis monooxygenase gene (AWC34_RS01805) identified in KM1031 as the possible determinants of chloramphenicol and erythromycin resistance. Nguyen and Nguyen^35^ reported that an *E*. *coli* transformant containing antibiotic ABC transporter ATP-binding protein homolog showed resistance to cefalotin, kanamycin, ampicillin, erythromycin and chloramphenicol. In addition, the antibiotic ABC transporter ATP-binding protein was reported to confer resistance to several antibiotics via a ribosomal protection mechanism^36^. Therefore, we assumed that the antibiotic ABC transporter ATP-binding protein gene may confer the chloramphenicol and erythromycin resistance to strain KM1031. However, the existence of an antibiotic ABC transporter ATP-binding protein gene (SEQU_RS24115) does not confer resistance to chloramphenicol and erythromycin to the strain UMC-CNS-924. The putative antibiotic ABC transporter ATP-binding protein of strain KM1031 showed 100% sequence identity to that of UMC-CNS-924 (Supplementary Table S4), which means that the homologs do not contribute to the chloramphenicol and erythromycin resistance if their transcriptional regulators function properly. Uniquely, an antibiotic biosynthesis monooxygenase gene (AWC34_RS01805) was identified only in strain KM1031; this gene was reported to alter polyketide antibiotics such as macrolide antibiotics through oxidation^37, 38^. Meanwhile, strain-specific possession of ABC transporter ATP-binding protein or antibiotic biosynthesis monooxygenase homologs in the 15 strains from cheeses hampered clarification of which gene determines the resistance (Supplementary Table S3). Interestingly, two IS6 family transposase genes (COG AWC34_RS01810 and AWC34_RS01800) were identified in the flanking regions of the antibiotic biosynthesis monooxygenase gene for strain KM1031. Antibiotic biosynthesis monooxygenase gene was also found in the contigs of strains RE2.35 and 908_10 and an IS6 family transposase gene (A4A32_12915) was identified upstream of the homolog in strain RE2.35. The contig of strain RE2.35 composed of a pseudogene exists between the transposase and antibiotic biosynthesis monooxygenase genes. The contig of strain 908_10 composed of a pseudogene and antibiotic biosynthesis monooxygenase gene suggests the existence of transposase in the flanking region. Therefore, we cautiously assumed that AWC34_RS01805 in KM1031 inserted by the action of transposase conferred the chloramphenicol and erythromycin resistance in this strain.

The penicillin G-resistant strain KM1031 harbours a plasmid encoding a putative β-lactamase gene (Table 2, Fig. 3). β-Lactamase homologs are found in the chromosomes of the other four strains, except strain KS1039. The putative amino acid sequence of the plasmid-encoded β-lactamase gene (AWC34_RS13020) shows 79.7–95.3% identity with those from homologs encoded chromosomally (Supplementary Table S4). Meanwhile, the plasmid-encoded β-lactamase has 97% identity with the Zn-dependent hydrolase of *S*. *aureus* (Supplementary Table S5). This suggests that the chromosomal β-lactamase homologs contribute not to penicillin G resistance but to other functions. The absence of a β-lactamase homolog in strain KS1039 implies that this gene is not critical to the survival of this strain.

Strain UMC-CNS-924 was resistant to tetracycline and was shown to contain a plasmid-encoded gene whose product has 99% sequence identity with tetracycline resistance MFS (major facilitator superfamily) efflux pumps of *Staphylococcus* species (Table 2, Fig. 3, Supplementary Table S5). This gene may thus encode an efflux pump that directly promotes resistance to tetracycline.

It is already well known that the *mecA* gene, which encodes the low-affinity penicillin-binding protein PBP 2A, confers methicillin resistance^39^. The *mecA* gene was not identified in the six strains, and methicillin resistance was also not identified. Meanwhile, three genes annotated to encode methicillin resistance proteins were commonly identified in the six strains. Two putative methicillin resistance proteins show ≥88% sequence identity with the aminoacyltransferase of *Staphylococcus* species (Supplementary Table S5). For these two genes, there is the possibility of mis-annotation. The third gene has ≥66% sequence identity with FemA of the *femAB* operon. FemA and FemB are known as additional components for methicillin resistance, which enhance the methicillin resistance of MecA. Therefore, these three genes may not confer methicillin resistance to *S*. *equorum* strains.

In the current study, all plasmid-mediated antibiotic resistance genes were linked to antibiotic resistance. However, most of the putative antibiotic resistance genes encoded chromosomally were not linked to their expected resistance. The potential antibiotic resistance genes encoded chromosomally may not contribute to resistance owing to their low activity^40, 41^. It is already well known that plasmid-mediated antibiotic resistance genes confer higher resistance than genes on chromosomes^42, 43^. However, antibiotic-resistant strains with chromosome-generated adaptation have been reported to survive at a higher rate than strains without antibiotic resistance genes upon exposure to antibiotics^41, 44^. Therefore, antibiotic resistance genes on chromosomes may allow organisms to survive under various conditions.

#### Two-component systems

The success of *S*. *aureus* as a pathogen is in part due to the precise regulation of genes for survival in various environments. Two-component systems (TCSs) serve as a basic stimulus-response coupling mechanism to allow organisms to sense and respond to changes in the environment. *S*. *aureus* has been reported to possess 16 TCSs within its relatively small genome, two of which, *saeRS* and *agrCA*, are known to regulate virulence^45–47^. In the genomes of *S*. *equorum* strains, three types of putative TCS involved in the regulation of ion acquisition, cell-wall synthesis and nitrate reduction have been identified (Table 3). *arlSR* (*yhcSR*) was reported to be essential for survival under various environmental conditions^48^ but was not reported to be involved in the regulation of virulence factors. *arlSR* TCS was also reported to regulate the biofilm formation of *Staphylococcus epidermidis* in an *ica*-dependent manner^49^. However, the *ica* gene involved in biofilm formation was not identified in the six *S*. *equorum* genomes. These results imply that *S*. *equorum arlSR* is involved not in the regulation of virulence genes but in adaptation to the environment. As another example, the *vraSR* TCS system was reported to be a positive modulator of cell wall biosynthesis^50^. Other TCSs in *S*. *equorum* have not been reported to be related to virulence factors. In this context, *S*. *equorum* may have little possibility of expressing the pathogenicity seen in *S*. *aureus*, but it can exhibit virulence simply by acquiring virulence determinants including those for haemolysis and antibiotic resistance.

**Table 3:** Putative two-component systems identified in six *S*. *equorum* genomes.

Function	Product	Gene	Gene locus					
			KM1031	C2014	KS1039	Mu2	UMC-CNS-924	G8HB1
Ion acquisition	Histidine kinase	*arlS*	AWC34_RS05855	AVJ22_RS06100	SE1039_RS06290	SEQMU2_RS11400	SEQU_RS19660	UF72_RS04635
	Response regulator	*arlR*	AWC34_RS05860	AVJ22_RS06105	SE1039_RS06295	SEQMU2_RS11405	SEQU_RS19665	UF72_RS04640
	Histidine kinase	*arlS*	AWC34_RS12210			SEQMU2_RS04475	SEQU_RS18135	UF72_RS08085
	Response regulator	*arlR*	AWC34_RS12205			SEQMU2_RS04470	SEQU_RS18130	UF72_RS08090
Cell wall synthesis	Sensor kinase	*vraS*	AWC34_RS08090	AVJ22_RS08380	SE1039_RS08510	SEQMU2_RS00385	SEQU_RS24695	UF72_RS12815
	Response regulator	*vraR*	AWC34_RS08085	AVJ22_RS08375	SE1039_RS08505	SEQMU2_RS00380	SEQU_RS24690	UF72_RS12810
Nitrate reduction	Histidine kinase		AWC34_RS07785	AVJ22_RS08060	SE1039_RS08200	SEQMU2_RS13330	SEQU_RS26235	UF72_RS13400
	Response regulator		AWC34_RS07780	AVJ22_RS08055	SE1039_RS08195	SEQMU2_RS13325	SEQU_RS26230	UF72_RS13405

### Salt tolerance of C2014 and KS1039

Bacteria respond to hyperosmotic stress either by controlling the flux of ions across their cellular membrane or by accumulating osmolytes called compatible solutes^51^. In ion homeostasis, potassium plays a pivotal role and is the most abundant ion in the cytoplasm of bacteria^52^. Although CNS are frequently identified in foods with a high salt concentration, the mechanism behind their salt tolerance is not well understood. Conversely, a number of characteristics allowing *S*. *aureus* to survive osmotic stress have been reported. For example, osmoprotectants such as choline, glycine, betaine and proline accumulate in *S*. *aureus* in response to osmotic stress^53, 54^. In addition, multiple genes, including the branched-chain amino acid transporter gene *brnQ* ^55^ and the arsenic operon regulatory gene *arsR* ^56^, have been reported to cooperate in conferring salt tolerance to *S*. *aureus*. Furthermore, the involvement of a very large cell-wall protein, Ebh, in the tolerance to transient hyperosmotic pressure was reported^57^. The phospholipid cardiolipin, an important component of the cell membrane, was also reported to be necessary for the prolonged survival of *S*. *aureus* in high-salt conditions^58, 59^. The six *S*. *equorum* strains also possess two cardiolipin synthetase genes (Supplementary Table S6), as well as a sodium/potassium transport system (Supplementary Table S7), and all strains exhibited growth on TSA plates supplemented with 15% (w/v) NaCl. Meanwhile, two strains, KS1039 and C2014, exhibited growth at a NaCl concentration of 25%; the growth rate of KS1039 was slightly higher than that of C2014 under these conditions (Supplementary Fig. S3).

Strain C2014 possesses an ortholog (AVJ22_RS01775) of an ion transporter that encodes a protein having a ball domain and a potassium voltage-gated channel in its ORF (Supplementary Fig. S4, Supplementary Table S7). The ball domain was shown to be responsible for the inactivation of voltage-gated ion channels^60, 61^. DNA sequences encoding ball domains have been widely identified in *Staphylococcus* species as well as in a *Bacillus* species and its relatives; the potassium voltage-gated channel-encoding sequences are located in the downstream parts of these ball domains (Supplementary Fig. S4). Strain KS1039 possesses two unique orthologs, SE1039_RS01900 and SE1039_RS01905, which encode a potassium voltage-gated channel and a protein with a ball domain, respectively; we supposed that these genes contribute to the high salt tolerance of strain KS1039^16^. The ion selectivity of potassium voltage-gated channels was reported to be associated with a conserved sequence motif TVGYG located in a re-entrant loop present in-between two predicted transmembrane regions^62^. This conserved sequence was identified in both AVJ22_RS01775 and SE1039-RS01900 genes.

**Figure 4:**
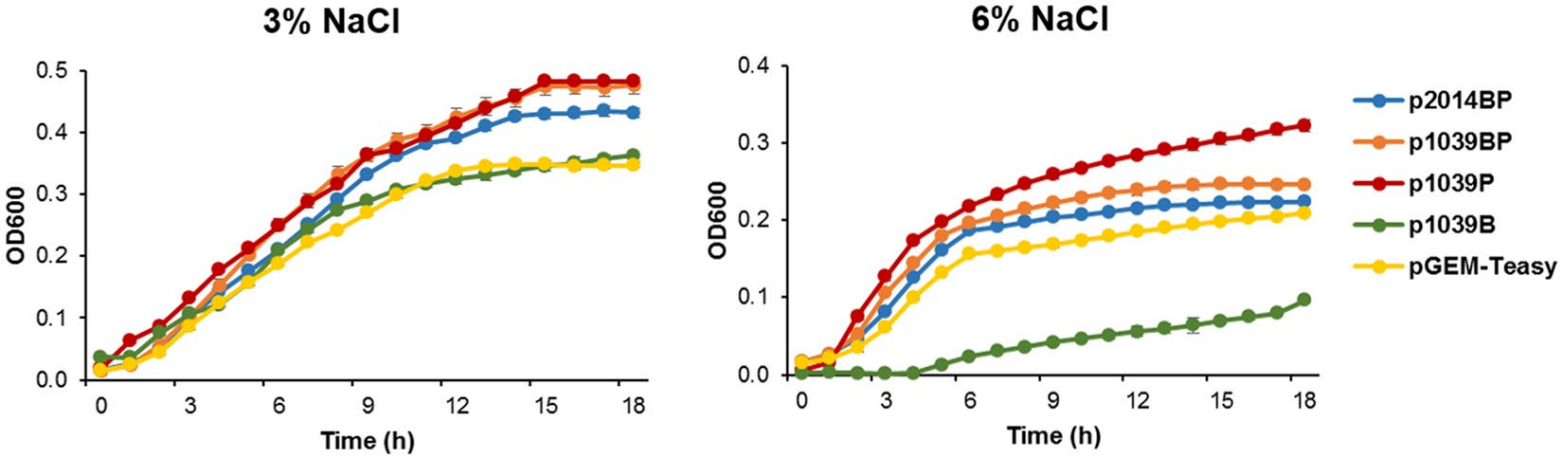
Effect of two types of potassium voltage-gated channel genes on the growth of *E*. *coli* cells under salt stress.

To investigate the effect of the potassium voltage-gated channel and ball domain on salt tolerance, the SE1039_RS01900/RS01905 and AVJ22_RS01775 genes were amplified and then cloned into the pGEM-T easy vector under the control of the T7 promoter. The resulting plasmids were designated as p1039P for the gene SE1039_RS01900, p1039B for the gene SE1039_RS01905, p1039BP for the genes SE1039_RS01905/RS01900 and p2014BP for the gene AVJ22_RS01775 (Supplementary Fig. S4). Under IPTG induction, the effect of NaCl on the growth was pronounced when NaCl was applied at a concentration of 6% (Fig. 4). The transformant harbouring p1039P showed the highest growth, followed by the transformant harbouring p1039BP. *E*. *coli* harbouring p1039B showed much slower growth than the control containing pGEM-T easy vector. These results suggest that the ball domain downregulates the activity of voltage-gated ion channels. Higher salt tolerances were exhibited in strain KS1039 and the transformant harbouring p1039BP than in strain C2014 and the recombinant harbouring p2014BP, implying that salt tolerance can be increased when potassium voltage-gated channels and ball domains exist in separate ORFs.

The flanking regions of the potassium voltage-gated channel genes for strains C2014 and KS1039 do not provide any clues about if or where any insertion might have occurred. However, transposase genes are found at distant loci on both sides of the potassium voltage-gated channel genes (COG AVJ22_RS01765 and AVJ22_RS03035 in strain C2014; SE1039_RS03265 and SE1039_RS01160 in strain KS1039). We thus assume that strains C2014 and KS1039 acquired the potassium voltage-gated channel gene by a random insertion event, enabling them to survive in the high-salt conditions of jeotgal. The potassium voltage-gated channel gene allows *S*. *equorum* strains to survive in high-salt fermented foods with a NaCl concentration of over 15%.

## Conclusions

The previously reported analyses of the three genomes of *S*. *equorum* strains Mu2, UMC-CNS-924 and KS1039 revealed that these three strains do not possess any clear virulence factors found in the well-known pathogen *S*. *aureus* ^10, 16, 17^. Genomic analysis should help to confirm the lack of known safety hazard determinants in strains, but cannot guarantee the safety of candidate industrial strains. This comparative genomic analysis identified the strain-specific determinants of *S*. *equorum* strains, which are linked to phenotypic characteristics including virulence and salt tolerance. The identification of factors related to phenotypic safety hazards should help in guaranteeing the safety of industrial strains. In this context, this study strongly supports the safety of strain KS1039, which was selected as a starter candidate for jeotgal fermentation^13^ because it does not possess any of the functional virulence determinants identified in the other strains. Additionally, this study identified the gene that contributes to the strain-specific high-salt tolerance of *S*. *equorum* strains. This study supports the usefulness of comparative genomics in the safety assessment and functional analysis of industrial strains including starter candidates for food fermentation.

## Electronic supplementary material

Supplementary Information
