# Supplementary material for: Genomic insights into the virulence and salt tolerance of Staphylococcus equorum
Source: Sci Rep. 2017 Jul 14;7:5383. doi: 10.1038/s41598-017-05918-5 (PMC5511256; doi:10.1038/s41598-017-05918-5)

Genomic insights into the virulence and salt tolerance of *Staphylococcus equorum*

Supplementary Information

Do-Won Jeong[1], Sojeong Heo[2], Sangryeol Ryu[3], Jochen Blom[4], Jong-Hoon Lee[2,*]

[1]Department of Food and Nutrition, Dongduk Women's University, Seoul 02748, Republic of Korea

[2]Department of Food Science and Biotechnology, Kyonggi University, Suwon 16227, Republic of Korea

[3]Department of Food and Animal Biotechnology, Department of Agricultural Biotechnology, and Center for Food and Bioconvergence, Seoul National University, Seoul 08826, Republic of Korea

[4] Bioinformatics and System Biology, Justus-Liebig-University Giessen, 35392 Giessen, Germany

*Corresponding author: Jong-Hoon Lee (email: jhl@kgu.ac.kr)

Table S1. Primer lists for the cloning of potassium voltage-gated channel genes.

**Target gene**	**Primer name**	**Sequence (5′→3′)**
AVJ22_RS01775	F-233	CCATTGGCGCGCCACAAATT
	R-234	GCTCGCAATAATCAATCA
SE1039_RS01900	F-217	CATTGGCGTAGCACAAAAGC
	R-250	CAATTAGCTAGATTCGCGC
SE1039_RS01905	F-251	GCGCGAATCTAGCTAATTG
	R-218	GCTCGGGATAACCAGACA
SE1039_RS01900 & SE1039_RS01905	F-217	CATTGGCGTAGCACAAAAGC
	R-218	GCTCGGGATAACCAGACA

Table S2. List of singletons generated by comparing the genomes of five *S. equorum* strains.

Strain	Gene locus	Product
KM1031	AWC34_RS00835	hypothetical protein
	AWC34_RS01535	UDP-glucose 4-epimerase
	AWC34_RS01540	capsular polysaccharide biosynthesis protein Cap 8F
	AWC34_RS01545	UDP-N-acetylglucosamine 2-epimerase
	AWC34_RS01550	O-acetyl transferase
	AWC34_RS01555	capsular polysaccharide biosynthesis protein
	AWC34_RS01560	hypothetical protein
	AWC34_RS01565	capsular biosynthesis protein
	AWC34_RS01570	UDP-N-acetyl-D-mannosamine dehydrogenase
	AWC34_RS01575	glycosyltransferase family 1
	AWC34_RS01585	hypothetical protein
	AWC34_RS01660	hypothetical protein
	AWC34_RS01805	antibiotic biosynthesis monooxygenase
	AWC34_RS01965	hypothetical protein
	AWC34_RS04410	hypothetical protein
	AWC34_RS11775	hypothetical protein
	AWC34_RS12575	type I site-specific deoxyribonuclease
	AWC34_RS12585	hypothetical protein
	AWC34_RS12600	hypothetical protein
	AWC34_RS12605	DNA methyltransferase
	AWC34_RS12610	restriction endonuclease
	AWC34_RS12655	hypothetical protein
	AWC34_RS12660	hypothetical protein
	AWC34_RS12665	hypothetical protein
	AWC34_RS13000	hypothetical protein
	AWC34_RS13030	hypothetical protein
	AWC34_RS13035	hypothetical protein
	AWC34_RS13040	hypothetical protein
	AWC34_RS12870	hypothetical protein
	AWC34_RS12875	hypothetical protein
	AWC34_RS12880	hypothetical protein
	AWC34_RS12925	hypothetical protein
	AWC34_RS12965	MFS transporter
	AWC34_RS12970	MFS transporter
	AWC34_RS12975	hypothetical protein
	AWC34_RS12985	hypothetical protein
	AWC34_RS12990	DNA-binding protein
	AWC34_RS13050	hypothetical protein
	AWC34_RS13170	4-hydroxy-tetrahydrodipicolinate synthase
	AWC34_RS13175	hypothetical protein
	AWC34_RS13190	hypothetical protein
	AWC34_RS13195	hypothetical protein
	AWC34_RS13195	hypothetical protein
	AWC34_RS13195	hypothetical protein
	AWC34_RS13195	hypothetical protein

Strain	Gene locus	Product
	AWC34_RS13200	hypothetical protein
	AWC34_RS13200	methyltryptophan oxidase
	AWC34_RS13230	RNA-directed DNA polymerase
	AWC34_RS13110	hypothetical protein
	AWC34_RS13245	hypothetical protein
	AWC34_RS13160	hypothetical protein
	AWC34_RS13170	4-hydroxy-tetrahydrodipicolinate synthase
	AWC34_RS13175	hypothetical protein
	AWC34_RS13190	hypothetical protein
	AWC34_RS13195	hypothetical protein
	AWC34_RS13200	methyltryptophan oxidase
	AWC34_RS13215	hypothetical protein
	AWC34_RS13230	hypothetical protein
	AWC34_RS13240	hypothetical protein
	AWC34_RS13160	hypothetical protein
C2014	AVJ22_RS00045	membrane protein
	AVJ22_RS00050	hypothetical protein
	AVJ22_RS00095	alpha-N-arabinofuranosidase
	AVJ22_RS00100	hypothetical protein
	AVJ22_RS00140	hypothetical protein
	AVJ22_RS00150	glycosyl transferase
	AVJ22_RS00160	hypothetical protein
	AVJ22_RS00465	linear gramicidin dehydrogenase LgrE
	AVJ22_RS13245	IS1272, transposase
	AVJ22_RS00545	hypothetical protein
	AVJ22_RS00575	hypothetical protein
	AVJ22_RS00580	hypothetical protein
	AVJ22_RS00585	myo-inosose-2 dehydratase
	AVJ22_RS00590	hypothetical protein
	AVJ22_RS00595	hypothetical protein
	AVJ22_RS00600	methylmalonate-semialdehyde dehydrogenase
	AVJ22_RS00605	hypothetical protein
	AVJ22_RS00610	5-dehydro-2-deoxygluconokinase
	AVJ22_RS00615	hypothetical protein
	AVJ22_RS00660	hypothetical protein
	AVJ22_RS01520	hypothetical protein
	AVJ22_RS01525	galactosamine-containing minor teichoic acid bio synthesis protein
	AVJ22_RS01535	hypothetical protein
	AVJ22_RS01705	hypothetical protein
	AVJ22_RS01710	hypothetical protein
	AVJ22_RS01735	hypothetical protein
	AVJ22_RS01745	hypothetical protein
	AVJ22_RS01750	hypothetical protein
	AVJ22_RS01755	(2-aminoethyl)phosphonate-pyruvate transaminase
	AVJ22_RS01760	hypothetical protein
	AVJ22_RS01780	hypothetical protein

Strain	Gene locus	Product
	AVJ22_RS01795	hypothetical protein
	AVJ22_RS01875	hypothetical protein
	AVJ22_RS02955	hypothetical protein
	AVJ22_RS03910	IS1272, transposase
	AVJ22_RS04345	integrase
	AVJ22_RS04350	hypothetical protein
	AVJ22_RS04355	hypothetical protein
	AVJ22_RS04360	hypothetical protein
	AVJ22_RS04370	hypothetical protein
	AVJ22_RS04375	hypothetical protein
	AVJ22_RS04380	hypothetical protein
	AVJ22_RS04385	hypothetical protein
	AVJ22_RS04395	hypothetical protein
	AVJ22_RS04395	cell division protein ZipA
	AVJ22_RS04785	transposase
	AVJ22_RS05345	hypothetical protein
	AVJ22_RS05340	hypothetical protein
	AVJ22_RS05355	hypothetical protein
	AVJ22_RS05375	hypothetical protein
	AVJ22_RS05395	hypothetical protein
	AVJ22_RS05400	hypothetical protein
	AVJ22_RS05420	hypothetical protein
	AVJ22_RS05425	hypothetical protein
	AVJ22_RS05435	hypothetical protein
	AVJ22_RS05445	hypothetical protein
	AVJ22_RS05450	dUTP pyrophosphatase
	AVJ22_RS05455	hypothetical protein
	AVJ22_RS05465	hypothetical protein
	AVJ22_RS05475	hypothetical protein
	AVJ22_RS05480	hypothetical protein
	AVJ22_RS05480	hypothetical protein
	AVJ22_RS05490	hypothetical protein
	AVJ22_RS05545	hypothetical protein
	AVJ22_RS05620	hypothetical protein
	AVJ22_RS05625	hypothetical protein
	AVJ22_RS05660	putative protein
	AVJ22_RS05820	hypothetical protein
	AVJ22_RS05825	lantibiotic dehydratase, N-terminal domain protein
	AVJ22_RS05830	lantibiotic dehydratase, C-terminal domain protein
	AVJ22_RS05835	hypothetical protein
	AVJ22_RS06375	hypothetical protein
	AVJ22_RS06845	hypothetical protein
	AVJ22_RS06850	hypothetical protein
	AVJ22_RS06855	hypothetical protein
	AVJ22_RS06860	hypothetical protei
	AVJ22_RS07210	hypothetical protein

Strain	Gene locus	Product
	AVJ22_RS08070	transposase
	AVJ22_RS08075	hypothetical protein
	AVJ22_RS08635	hypothetical protein
	AVJ22_RS08660	hypothetical protein
	AVJ22_RS08665	hypothetical protein
	AVJ22_RS13245	IS1272, transposase
	AVJ22_RS09905	transposase
	AVJ22_RS10620	hypothetical protein
	AVJ22_RS13245	transposase
	AVJ22_RS12225	transposase
	AVJ22_RS12500	hypothetical protein
	AVJ22_RS12775	oxidoreductase, NAD-binding domain protein
	AVJ22_RS12780	hydrolase
	AVJ22_RS12785	hypothetical protein
	AVJ22_RS12790	hypothetical protein
	AVJ22_RS12915	hypothetical protein
	AVJ22_RS12920	hypothetical protein
	AVJ22_RS13005	leucine dehydrogenase
	AVJ22_RS13015	hypothetical protein
	AVJ22_RS13280	putative plasmid partition protein
	AVJ22_RS13285	CopG family transcriptional regulator
	AVJ22_RS13290	hypothetical protein
	AVJ22_RS13295	hypothetical protein
	AVJ22_RS13300	hypothetical protein
	AVJ22_RS13305	hypothetical protein
	AVJ22_RS13310	hypothetical protein
	AVJ22_RS13315	hypothetical protein
	AVJ22_RS13015	hypothetical protein
	AVJ22_RS13420	transposase
	AVJ22_RS13425	transposase
	AVJ22_RS13430	GntR family transcriptional regulator
	AVJ22_RS13435	hypothetical protein
	AVJ22_RS13440	hypothetical protein
	AVJ22_RS13445	tagatose-bisphosphate aldolase
	AVJ22_RS13450	beta-galactosidase
	AVJ22_RS13455	PTS mannose transporter subunit IIAB
	AVJ22_RS13460	hypothetical protein
	AVJ22_RS13465	hypothetical protein
	AVJ22_RS13470	hypothetical protein
	AVJ22_RS13480	transporter
	AVJ22_RS13495	hypothetical protein
	AVJ22_RS13530	hypothetical protein
	AVJ22_RS13535	6-carboxyhexanoate-CoA ligase
	AVJ22_RS13540	hypothetical protein
	AVJ22_RS13550	adenosylmethionine-8-amino-7-oxononanoate aminotransferase

Strain	Gene locus	Product
	AVJ22_RS13565	adenosylmethionine-8-amino-7-oxononanoate aminotransferase
	AVJ22_RS13565	dethiobiotin synthase
	AVJ22_RS13565	membrane protein
	AVJ22_RS13570	hypothetical protein
	AVJ22_RS13580	hypothetical protein
	AVJ22_RS13590	hypothetical protein
	AVJ22_RS13600	hypothetical protein
	AVJ22_RS13625	sn-glycerol-3-phosphate transporter
	AVJ22_RS13640	putative protein
	AVJ22_RS13770	hypothetical protein
	AVJ22_RS13775	ribonuclease H
	AVJ22_RS13785	hypothetical protein
	AVJ22_RS13790	hypothetical protein
	AVJ22_RS13795	hypothetical protein
	AVJ22_RS13800	hypothetical protein
	AVJ22_RS13805	hypothetical protein
	AVJ22_RS13825	hypothetical protein
	AVJ22_RS14005	hypothetical protein
	AVJ22_RS13640	putative protein
	AVJ22_RS14015	hypothetical protein
	AVJ22_RS14050	hypothetical protein
	AVJ22_RS14055	hypothetical protein
	AVJ22_RS14060	hypothetical protein
	AVJ22_RS14065	hypothetical protein
	AVJ22_RS14095	haemolysin family calcium-binding region
KS1039	SE1039_00170	hypothetical protein
	SE1039_00200	hypothetical protein
	SE1039_00300	phenolic acid decarboxylase PadC
	SE1039_00310	hypothetical protein
	SE1039_00380	hypothetical protein
	SE1039_00400	acyl esterase
	SE1039_00430	hypothetical protein
	SE1039_00440	Macrolide 2-phosphotransferase II
	SE1039_00480	GntR family transcriptional regulator
	SE1039_00490	3-isopropylmalate dehydrogenase
	SE1039_00500	cation transporter
	SE1039_00540	hypothetical protein
	SE1039_00570	hypothetical protein
	SE1039_00580	hypothetical protein
	SE1039_00590	ArsR family transcriptional regulator
	SE1039_00670	hypothetical protein
	SE1039_01890	hypothetical protein
	SE1039_01910	hypothetical protein
	SE1039_01920	hypothetical protein
	SE1039_01930	hypothetical protein

Strain	Gene locus	Product
	SE1039_01940	hypothetical protein
	SE1039_03050	hypothetical protein
	SE1039_03060	hypothetical protein
	SE1039_03200	hypothetical protein
	SE1039_03210	hypothetical protein
	SE1039_03220	hypothetical protein
	SE1039_03540	TetR family transcriptional regulator
	SE1039_03560	hypothetical protein
	SE1039_03570	phosphotransferase
	SE1039_03580	hypothetical protein
	SE1039_03590	hypothetical protein
	SE1039_03630	hypothetical protein
	SE1039_03640	hypothetical protein
	SE1039_03760	diguanylate cyclase
	SE1039_03780	NADP oxidoreductase
	SE1039_04010	hypothetical protein
	SE1039_09040	putative protein
	SE1039_09050	hypothetical protein
	SE1039_09070	hypothetical protein
	SE1039_09080	hypothetical protein
	SE1039_09090	hypothetical protein
	SE1039_09100	hypothetical protein
	SE1039_09120	hypothetical protein
	SE1039_09610	integrase
	SE1039_09620	hypothetical protein
	SE1039_09630	hypothetical protein
	SE1039_09660	hypothetical protein
	SE1039_09670	hypothetical protein
	SE1039_09680	hypothetical protein
	SE1039_09690	hypothetical protein
	SE1039_09700	hypothetical protein
	SE1039_09710	hypothetical protein
	SE1039_09720	hypothetical protein
	SE1039_09730	chromosome replication initiation protein DnaA
	SE1039_09740	hypothetical protein
	SE1039_09750	single-stranded DNA-binding protein
	SE1039_09760	hypothetical protein
	SE1039_09770	hypothetical protein
	SE1039_09780	hypothetical protein
	SE1039_09810	hypothetical protein
	SE1039_09820	RusA
	SE1039_09840	hypothetical protein
	SE1039_09910	putative protein
	SE1039_09950	terminase
	SE1039_09990	putative protein
	SE1039_10110	hypothetical protein

Strain	Gene locus	Product
	SE1039_10120	hypothetical protein
	SE1039_10140	putative protein
	SE1039_10210	chromosome partitioning protein ParA
	SE1039_10220	hypothetical protein
	SE1039_10230	hypothetical protein
	SE1039_10250	hypothetical protein
	SE1039_10540	hypothetical protein
	SE1039_11680	hypothetical protein
	SE1039_11690	hypothetical protein
	SE1039_11700	hypothetical protein
	SE1039_12000	hypothetical protein
	SE1039_12010	phenolic acid decarboxylase subunit B
	SE1039_12020	hypothetical protein
	SE1039_12030	hypothetical protein
	SE1039_12040	hypothetical protein
	SE1039_15930	hypothetical protein
	SE1039_17640	hypothetical protein
	SE1039_21600	hypothetical protein
	SE1039_23220	carbohydrate kinase
	SE1039_25290	membrane protein
	SE1039_25650	protein-Npi-phosphohistidine-sugar phosphotransferase
	SE1039_25660	hypothetical protein
	SE1039_25690	exporter protein
	SE1039_25700	hypothetical protein
	SE1039_25710	hypothetical protein
	SE1039_25720	RNA-directed DNA polymerase
	SE1039_25730	putative membrane protein
	SE1039_25740	hypothetical protein
	SE1039_25910	hypothetical protein
	SE1039_25960	hypothetical protein
	SE1039_25970	CRISPR-associated protein Cas6
	SE1039_25980	CRISPR-associated protein Csm6
	SE1039_25990	CRISPR-associated protein Csm5
	SE1039_26000	CRISPR-associated protein Csm4
	SE1039_26010	CRISPR-associated protein Cmr4
	SE1039_26020	CRISPR-associated protein Csm2
	SE1039_26030	CRISPR-associated protein Csm1
	SE1039_26040	CRISPR-associated Csm1 family protein
	SE1039_26050	CRISPR-associated protein Cas2
	SE1039_26060	CRISPR-associated protein Cas1
	SE1039_26070	restriction endonuclease subunit R
	SE1039_26080	hypothetical protein
	SE1039_26190	hypothetical protein
	SE1039_26240	hypothetical protein
	SE1039_26250	hypothetical protein
	SE1039_26270	hypothetical protein

Strain	Gene locus	Product
	SE1039_26320	CDP-glycerol glycerophosphotransferase
	SE1039_26410	hypothetical protein
	SE1039_26420	hypothetical protein
	SE1039_26490	hypothetical protein
	SE1039_26500	hypothetical protein
Mu2	SEQMU2_RS14270	hypothetical protein
	SEQMU2_RS14275	hypothetical protein
	SEQMU2_RS14280	hypothetical protein
	SEQMU2_RS14285	MerR family transcriptional regulator
	SEQMU2_RS14290	integrase
	SEQMU2_RS14295	hypothetical protein
	SEQMU2_RS14300	hypothetical protein
	SEQMU2_RS14305	hypothetical protein
	SEQMU2_RS14240	hypothetical protein
	SEQMU2_RS14260	hypothetical protein
	SEQMU2_RS14265	hypothetical protein
	SEQMU2_RS14075	Zn-dependent alcohol dehydrogenase
	SEQMU2_RS14105	hypothetical protein
	SEQMU2_RS14120	hypothetical protein
	SEQMU2_RS14180	hypothetical protein
	SEQMU2_RS13815	hypothetical protein
	SEQMU2_RS13820	hypothetical protein
	SEQMU2_RS13835	hypothetical protein
	SEQMU2_RS13840	addiction module protein
	SEQMU2_RS13845	prevent-host-death protein
	SEQMU2_RS13850	hypothetical protein
	SEQMU2_RS13855	hypothetical protein
	SEQMU2_RS13860	hypothetical protein
	SEQMU2_RS13885	hypothetical protein
	SEQMU2_RS13915	hypothetical protein
	SEQMU2_RS13925	transporter
	SEQMU2_RS13935	hypothetical protein
	SEQMU2_RS10665	hypothetical protein
	SEQMU2_RS10675	DNA-binding protein
	SEQMU2_RS10680	Rha family transcriptional regulator
	SEQMU2_RS10685	CRISPR-associated protein Cas2
	SEQMU2_RS10690	hypothetical protein
	SEQMU2_RS10695	hypothetical protein
	SEQMU2_RS10700	hypothetical protein
	SEQMU2_RS10705	hypothetical protein
	SEQMU2_RS10750	hypothetical protein
	SEQMU2_RS11280	hypothetical protein
	SEQMU2_RS11325	hypothetical protein
	SEQMU2_RS11330	hypothetical protein
	SEQMU2_RS11340	hypothetical protein
	SEQMU2_RS11345	hypothetical protein

Strain	Gene locus	Product
	SEQMU2_RS11350	hypothetical protein
	SEQMU2_RS11355	phage protein
	SEQMU2_RS11360	DNA primase
	SEQMU2_RS11365	hypothetical protein
	SEQMU2_RS11370	hypothetical protein
	SEQMU2_RS11375	hypothetical protein
	SEQMU2_RS11380	hypothetical protein
	SEQMU2_RS11385	phage integrase
	SEQMU2_RS12835	integrase
	SEQMU2_RS08255	pathogenicity island protein
	SEQMU2_RS08265	hypothetical protein
	SEQMU2_RS08270	hypothetical protein
	SEQMU2_RS08275	hypothetical protein
	SEQMU2_RS08280	hypothetical protein
	SEQMU2_RS08285	hypothetical protein
	SEQMU2_RS08295	hypothetical protein
	SEQMU2_RS08300	hypothetical protein
	SEQMU2_RS08585	5-aminolevulinate synthase
	SEQMU2_RS08225	DNA-binding protein
	SEQMU2_RS08230	antirepressor
	SEQMU2_RS08235	hypothetical protein
	SEQMU2_RS08240	hypothetical protein
	SEQMU2_RS08245	primase
	SEQMU2_RS08250	hypothetical protein
	SEQMU2_RS06750	hydrolase
	SEQMU2_RS07815	hypothetical protein
	SEQMU2_RS07820	hypothetical protein
	SEQMU2_RS07825	hypothetical protein
	SEQMU2_RS07830	repressor
	SEQMU2_RS07835	hypothetical protein
	SEQMU2_RS07845	phage protein
	SEQMU2_RS07850	hypothetical protein
	SEQMU2_RS07855	hypothetical protein
	SEQMU2_RS07860	hypothetical protein
	SEQMU2_RS07865	hypothetical protein
	SEQMU2_RS07870	hypothetical protein
	SEQMU2_RS07875	hypothetical protein
	SEQMU2_RS07880	hypothetical protein
	SEQMU2_RS07885	hypothetical protein
	SEQMU2_RS07890	single-stranded DNA-binding protein
	SEQMU2_RS07895	phage protein
	SEQMU2_RS07900	hypothetical protein
	SEQMU2_RS07910	hypothetical protein
	SEQMU2_RS07915	hypothetical protein
	SEQMU2_RS07920	hypothetical protein
	SEQMU2_RS07930	hypothetical protein

Strain	Gene locus	Product
	SEQMU2_RS07935	hypothetical protein
	SEQMU2_RS07940	phage protein
	SEQMU2_RS07950	hypothetical protein
	SEQMU2_RS07955	hypothetical protein
	SEQMU2_RS07995	terminase
	SEQMU2_RS08025	phage protein
	SEQMU2_RS08040	hypothetical protein
	SEQMU2_RS08050	hypothetical protein
	SEQMU2_RS08085	hypothetical protein
	SEQMU2_RS08090	hypothetical protein
	SEQMU2_RS06470	pathogenicity island protein
	SEQMU2_RS06485	hypothetical protein
	SEQMU2_RS06495	mRNA interferase PemK
	SEQMU2_RS06515	hypothetical protein
	SEQMU2_RS05430	hypothetical protein
	SEQMU2_RS05435	hypothetical protein
	SEQMU2_RS05440	hypothetical protein
	SEQMU2_RS05445	hypothetical protein
	SEQMU2_RS05450	hypothetical protein
	SEQMU2_RS05455	hypothetical protein
	SEQMU2_RS05770	membrane protein
	SEQMU2_RS06210	hypothetical protein
	SEQMU2_RS06215	hypothetical protein
	SEQMU2_RS06220	hypothetical protein
	SEQMU2_RS06425	integrase
	SEQMU2_RS06430	DNA-binding protein
	SEQMU2_RS06435	hypothetical protein
	SEQMU2_RS06440	hypothetical protein
	SEQMU2_RS06445	hypothetical protein
	SEQMU2_RS06450	hypothetical protein
	SEQMU2_RS06460	hypothetical protein
	SEQMU2_RS06465	hypothetical protein
	SEQMU2_RS05165	hypothetical protein
	SEQMU2_RS05170	hypothetical protein
	SEQMU2_RS05205	activator of HSP90 ATPase
	SEQMU2_RS05215	glutamine amidotransferase
	SEQMU2_RS05220	DeoR family transcriptional regulator
	SEQMU2_RS05225	hypothetical protein
	SEQMU2_RS05230	hypothetical protein
	SEQMU2_RS05235	hypothetical protein
	SEQMU2_RS05250	acetolactate synthase
	SEQMU2_RS05270	hypothetical protein
	SEQMU2_RS05275	hypothetical protein
	SEQMU2_RS05315	hypothetical protein
	SEQMU2_RS05320	hypothetical protein
	SEQMU2_RS05325	hypothetical protein

Strain	Gene locus	Product
	SEQMU2_RS05335	hypothetical protein
	SEQMU2_RS05355	capsular polysaccharide biosynthesis protein CapM
	SEQMU2_RS05370	capsule biosynthesis protein CapJ
	SEQMU2_RS05380	hypothetical protein
	SEQMU2_RS05385	capsular polysaccharide biosynthesis protein
	SEQMU2_RS05025	pathogenicity island protein
	SEQMU2_RS05035	abortive infection protein
	SEQMU2_RS05040	hypothetical protein
	SEQMU2_RS05045	hypothetical protein
	SEQMU2_RS05050	hypothetical protein
	SEQMU2_RS05060	hypothetical protein
	SEQMU2_RS05065	hypothetical protein
	SEQMU2_RS05075	phage head-tail adapter protein
	SEQMU2_RS05080	pathogenicity island protein
	SEQMU2_RS04905	hypothetical protein
	SEQMU2_RS04915	transcriptional regulator
	SEQMU2_RS04955	hypothetical protein
	SEQMU2_RS04595	Erm Leader peptide
	SEQMU2_RS04870	hypothetical protein
	SEQMU2_RS04875	hypothetical protein
	SEQMU2_RS02440	GntR family transcriptional regulator
	SEQMU2_RS02445	PTS beta-glucoside transporter subunit IIABC
	SEQMU2_RS00570	Linalool 8-monooxygenase
	SEQMU2_RS00575	hypothetical protein
	SEQMU2_RS00580	hydrolase
	SEQMU2_RS00280	recombinase
	SEQMU2_RS00285	phage protein
	SEQMU2_RS00290	DNA-binding protein
	SEQMU2_RS00295	excisionase
	SEQMU2_RS00300	hypothetical protein
	SEQMU2_RS00305	hypothetical protein
	SEQMU2_RS00310	DNA primase
	SEQMU2_RS00315	hypothetical protein
UMC-CNS-924	SEQU_RS16265	hypothetical protein
	SEQU_RS17065	hypothetical protein
	SEQU_RS17850	hypothetical protein
	SEQU_RS18240	hypothetical protein
	SEQU_RS18245	hypothetical protein
	SEQU_RS20935	hypothetical protein
	SEQU_RS20945	hypothetical protein
	SEQU_RS20950	hypothetical protein
	SEQU_RS20955	hypothetical protein
	SEQU_RS20960	hypothetical protein
	SEQU_RS20965	hypothetical protein
	SEQU_RS20970	hypothetical protein

Strain	Gene locus	Product
	SEQU_RS21005	hypothetical protein
	SEQU_RS21050	hypothetical protein
	SEQU_RS21440	membrane protein
	SEQU_RS23595	pyridoxal phosphate-dependent aminotransferase
	SEQU_RS23600	hypothetical protein
	SEQU_RS23610	capsular biosynthesis protein
	SEQU_RS23630	hypothetical protein
	SEQU_RS23635	hypothetical protein
	SEQU_RS23640	hypothetical protein
	SEQU_RS23645	hypothetical protein
	SEQU_RS25040	hypothetical protein
	SEQU_RS25215	hypothetical protein
	SEQU_RS25225	hypothetical protein
	SEQU_RS25230	hypothetical protein
	SEQU_RS25240	hypothetical protein
	SEQU_RS26430	hypothetical protein
	SEQU_RS26440	hypothetical protein
	SEQU_RS0113725	antibiotic biosynthesis monooxygenase
	SEQU_RS26470	hypothetical protein
	SEQU_RS26480	glycerol dehydrogenase
	SEQU_RS26495	hypothetical protein
	SEQU_RS26500	peptidase C39
	SEQU_RS26540	hypothetical protein
	SEQU_RS26600	recombinase
	SEQU_RS26605	CopG family transcriptional regulator
	SEQU_RS26610	replication initiation protein
	SEQU_RS26615	tetracycline resistance MFS efflux pump
	SEQU_RS26660	replication protein

Table S3. Potential virulence determinants identified in the genomes of 15 *S. equorum* strains from cheeses.

Virulence factor	Gene identification														
	White_ SAM	OffWhite _SAM	BC9	BC3	900_4	341_10	RE2.24	RE2.35	RE2.40	738_7	908_10	947_12	962_6	862_5	AR8-13
**Haemolysis-related**															
Haemolysin	●	●	●	●	●	●	●	●	●	●	●	●	●	●	●
Haemolysin III	●	●	●	●	●	●	●	●	●	●	●	●	●	●	●
Haemolysin activation protein	●	●	●	●	●	●	●	●	●	●	●	●	●	●	●
Haemolysin family calcium-binding region															
**Antibiotic resistance**															
Efflux pump															
Chloramphenicol resistance protein DHA1	●	●	●	●	●	●	●	●	●	●	●	●	●	●	●
Lincomycin resistance protein LmrB	●	●	●	●	●	●	●	●	●	●	●	●	●	●	●
Quinolone resistance protein NorB	●	●	●	●	●	●	●	●		●	●	●	●	●	●
Quinolone resistance protein NorB				●	●	●	●	●	●	●	●	●	●	●	●
Multidrug resistance protein SepA	●	●	●	●	●	●	●	●	●	●	●	●	●	●	●
Multidrug resistance protein SMR	●	●	●	●	●	●	●	●	●	●	●	●	●	●	●
Antibiotic ABC transporter ATP-binding protein				●			●	●		●	●	●	●	●	●
Tetracycline resistance MFS efflux pump															
Enzymatic inactivation															
Methicillin resistance protein	●	●	●	●	●	●	●	●	●	●	●	●	●	●	●
Methicillin resistance protein	●	●	●	●	●	●	●	●	●	●	●	●	●	●	●
Methicillin resistance protein FemA	●		●	●	●	●	●		●						●
β-Lactamase	●	●	●	●							●				
Lincomycin resistance protein LnuA															
Other															
Antibiotic biosynthesis monooxygenase								●			●				

Table S4**.** The putative amino acid identities between annotated potential virulence genes in *S. equorum*.

**Virulence factor**	**KM1031**	**C2014**	**KS1039**	**Mu2**	**UMC-CNS-924**	**G8HB1**
**Haemolysis-related**[a]						
Haemolysin	98.8	100	–	98.8	98.8	99.1
Haemolysin III	99.6	100	100	100	99.6	96.5
Haemolysin activation protein	97.7	100	97.7	97.7	30.2 (100)<br>37.2 (54.5)<br>30.2 (45.5)	97.7
Haemolysin family calcium-binding region		100				
**Antibiotic resistance**[b]						
Chloramphenicol resistance protein DHA1	100	98.7	99.7	99.7	99.5	100
Lincomycin resistance protein LmrB	100	97.0	–	99.1	98.9	98.9
Quinolone resistance protein NorB	100	97.8	99.3	99.3	100	100
Quinolone resistance protein NorB	100	41.5	98.9	44.3	99.8	99.6
Multidrug resistance protein SepA	100	99.3	100	100	100	98.0
Multidrug resistance protein SMR	100	98.1	99.1	100	100	100
Methicillin resistance protein	100	99.3	99.8	100	100	100
Methicillin resistance protein	100	99.8	99.5	99.5	99.8	99.8
Methicillin resistance protein femA	100	97.4	99.0	99.0	100	98.5
β-Lactamase	100	80.4	–	79.7	95.3	79.7
Lincomycin resistance protein LnuA	100	–	–	–	96.3	–
Tetracycline resistance MFS efflux pump	–	–	–	–	100	–
Antibiotic biosynthesis monooxygenase	100	–	–	–	–	–
Antibiotic ABC transporter ATP-binding protein	100	–	–	–	100	100

[a] The putative amino acid identities of haemolysis-related genes were compared with those from strain C2014. Identities in parentheses were calculated among the three haemolysin activation proteins identified in UMC-CNS-924 based on the gene SEQU_RS16015.

[b] Putative amino acid sequences from the virulence genes of strain KM1031 were used for identity determination, except in the case of tetracycline resistance MFS efflux pump gene, which was only identified in strain UMC-CNS-924.

Table S5. Putative antibiotic resistance factors identified in six *S. equorum* strains and their homologous proteins in other organisms.

Putative virulence factor	Strain	Homologous protein	Identity	Accession No.
Chloramphenicol resistance protein DHA1	*S. xylosus*	MFS transporter	89	WP_039067373.1
	S. succinus	MFS transporter	84	WP_046835850.1
	S. sapophyticus	chloramphenicol resistance protein	85	WP_002482402.1
	S. haemolyticus	MFS transporter	74	WP_049410867.1
	S. aureus	MFS transporter	72	WP_000115415.1
Lincomycin resistance protein LmrB	*S. sapophyticus*	MFS transporter	87	WP_011302025.1
	Streptococcus equi	major facilitator superfamily permease	86	CRV27957.1
	S. cohnii	MFS transporter	85	WP_040030061.1
	S. xylosus	MFS transporter	83	WP_039069546.1
	S. succinus	MFS transporter	80	WP_046837658.1
Quinolone resistance protein NorB	*S. xylosus*	MFS transporter	83	WP_039067198.1
	S. cohnii	MFS transporter	79	WP_046207151.1
	S. succinus	MFS transporter	76	WP_046836975.1
	S. gallinarum	MFS transporter	76	WP_046836975.1
	S. simiae	MFS transporter	64	WP_050801492.1
Quinolone resistance protein NorB	*S. xylosus*	MFS transporter	89	WP_039069313.1
	S. cohnii	MFS transporter	87	WP_040029752.1.
	S. succinus	MFS transporter	86	WP_046835963.1
	S. gallinarum	MFS transporter	82	WP_042740359.1
	S. microti	MFS transporter	80	WP_044360081.1
Multidrug resistance protein SepA	*S. gallinarum*	multidrug resistance protein SepA	94	WP_042739039.1
	S. xylosus	multidrug resistance protein SepA	93	WP_029378551.1
	S. cohnii	multidrug resistance protein SepA	91	WP_046208389.1
	S. saprophyticus	multidrug resistance protein SepA	90	WP_041080835.1
	S. succinus	multidrug resistance protein SepA	88	WP_046835623.1
Multidrug resistance protein SMR	*Staphylococcus*	multidrug resistance protein SMR	100	WP_002506070.1
	Staphylococcus sp.	multidrug resistance protein SMR	97	WP_057511639.1
	S. xylosus	multidrug resistance protein SMR	81	WP_039069244.1

Putative virulence factor	Strain	Homologous protein	Identity	Accession No.
	S. succinus	multidrug resistance protein SMR	80	WP_046836909.1
	S. cohnii	multidrug resistance protein SMR	78	WP_064209634.1
Methicillin resistance protein	*S. xylosus*	aminoacyltransferase	93	WP_039066581.1
	S. succinus	aminoacyltransferase	91	WP_046837466.1
	S. xylosus	factor essential for methicillin resistance FemA	91	AAC69634.1
	S. cohnii	aminoacyltransferase	89	WP_046208312.1
	S. gallinarum	aminoacyltransferase	88	WP_042738736.1
Methicillin resistance protein	*S. saprophyticus*	aminoacyltransferase	93	WP_011303147.1
	S. xylosus	aminoacyltransferase	93	WP_047172408.1
	S. gallinarum	aminoacyltransferase	91	WP_042738735.1
	S. succinus	aminoacyltransferase	90	WP_046837465.1
	S. cohnii	aminoacyltransferase	90	WP_046208311.1
Methicillin resistance protein FemA	*S. epidermidis*	FemA protein of FemAB family	67	ADA62617.1
	Bacillales	methicillin resistance protein	67	WP_020364758.1
	S. epidermidis	methicillin resistance protein	66	WP_064206559.1
	S. condimenti	methicillin resistance protein	66	WP_047132321.1
	S. aureus	meticillin resistance protein	66	WP_047210510.1
β-Lactamase	*S. aureus*	Zn-dependent hydrolase	97	WP_031885496.1
	S. arlettae	Zn-dependent hydrolase	97	WP_002510745.1
	S. saprophyticus	Zn-dependent hydrolase	96	WP_041080736.1
	S. vitylinus	hypothetical protein	96	WP_016911205.1
Lincomycin resistance protein LnuA	*S. hemoliticus*	lincosamide nucleotidyltransferase	99	WP_053020242.1
	Lactobacillus oryzae	lincosamide nucleotidyltransferase	99	WP_034529812.1
	S. aureus	lincosamide nucleotidyltransferase	96	WP_0.3491106.1
Tetracycline resistance MFS efflux pump [a]	*Bacilli*	tetracycline resistance MFS efflux pump	100	WP_000492283.1
	S. aureus	tetracycline resistance protein	99	EVJ05357.1
	S. epidermidis	tetracycline resistance MFS efflux pump	99	WP_040118612.1
	S. aureus	tetracycline resistance MFS efflux pump	99	WP_000492285.1
	S. epidermidis	tetracycline efflux MFS transporter TetK	99	WP_064658524.1
Antibiotic biosynthesis monooxygenase	*S. saprophyticus*	antibiotic biosynthesis monoxygenase	100	WP_002484388.1

Putative virulence factor	Strain	Homologous protein	Identity	Accession No.
	Jeotgalicoccus psychrophilus	antibiotic biosynthesis monoxygenase	99	WP_026859823.1
	S. xylosus	antibiotic biosynthesis monoxygenase	99	WP_017723728.1
	Macrococcus caseolyticus	antibiotic biosynthesis monoxygenase	96	WP_012655943.1
	Aquinocola tertiaricarbonis	antibiotic biosynthesis monoxygenase	78	WP_046111372.1
Antibiotic ABC transporter ATP-binding protein	*S. aureus*	ABC-F type ribosomal protection protein	77	WP_031881658.1
	Listeria monocytogenes	macrolide efflux ABC Msr(A)	76	WP_063455884.1
	S. epidermidis	Msr family ABC-F type ribosomal protection protein	74	WP_049385452.1
	S. cohnii	macrolide efflux ABC Msr(A)	74	WP_040030594.1
	Pseudomonas sp.	ABC-F type ribosomal protection protein Msr(A)	74	WP_063854341.1

The putative amino acid sequences of antibiotic resistance factors from strain KM1031 were used for identity determination, except in the case of tetracycline resistance MFS efflux pump only identified in strain UMC-CNS-924.

Table S6. Putative cardiolipin genes identified in six *S. equorum* genomes.

Gene	Gene locus					
	**KM1031**	**C2014**	**KS1039**	**Mu2**	**UMC-CNS-924**	**G8HB1**
Cardiolipin synthetase	AWC34_RS11580	AVJ22_RS11875	SE1039_RS11995	SEQMU2_RS03840	SEQU_RS17505	UF72_RS11685
Cardiolipin synthetase	AWC34_RS08615	AVJ22_RS08965	SE1039_RS09040	SEQMU2_RS00915	SEQU_RS25860	UF72_RS11685

Table S7**.** Sodium/potassium transport systems in six *S. equorum* strains

**Sodium/potassium efflux system**	**KM1031**	**C2014**	**KS1039**	**Mu2**	**UMC-CNS-924**	**G8HB1**
Potassium efflux system KefA protein	AWC34_RS00590	AVJ22_RS00505	SE1039_RS00665	SEQMU2_RS06175	SEQU_RS22955	UF72_RS06855
Potassium voltage-gated channel subfamily KQT		AVJ22_RS01775	SE1039_RS01900			
Potassium voltage-gated channel subfamily KQT			SE1039_RS01905			
Potassium uptake protein, integral membrane component, KtrB	AWC34_RS03930	AVJ22_RS03725	SE1039_RS03890	SEQMU2_RS09120	SEQU_RS22200	UF72_RS02425
Trk system potassium uptake protein TrkA	AWC34_RS04230	AVJ22_RS04060	SE1039_RS04235	SEQMU2_RS09445	SEQU_RS15715	UF72_RS02725
Glutathione-regulated potassium-efflux system ATP-binding protein	AWC34_RS02815	AVJ22_RS02605	SE1039_RS02765	SEQMU2_RS07555	SEQU_RS21575	UF72_RS01275
Glutathione-regulated potassium-efflux system ATP-binding protein	AWC34_RS05695	AVJ22_RS05960	SE1039_RS06120	SEQMU2_RS11170	SEQU_RS19500	UF72_RS04485
Glutathione-regulated potassium-efflux system ATP-binding protein	AWC34_RS06795		SE1039_RS07225	SEQMU2_RS12335	SEQU_RS19295	UF72_RS05575
Glutathione-regulated potassium-efflux system ATP-binding protein	AWC34_RS08420	AVJ22_RS08760	SE1039_RS08840	SEQMU2_RS00730	SEQU_RS26055	UF72_RS13145
Sodium-dependent transporter	AWC34_RS00935	AVJ22_RS00915	SE1039_RS01045	SEQMU2_RS06630	SEQU_RS23300	UF72_RS06515
Sodium:alanine symporter	AWC34_RS02090	AVJ22_RS01880	SE1039_RS02045	SEQMU2_RS06830	SEQU_RS22580	UF72_RS00550
Sodium:alanine symporter	AWC34_RS03880	AVJ22_RS03675	SE1039_RS03840	SEQMU2_RS09070	SEQU_RS22150	UF72_RS02375
Sodium:alanine symporter	AWC34_RS05500	AVJ22_RS05725	SE1039_RS05885	SEQMU2_RS10980	SEQU_RS16980	UF72_RS04290
Sodium:dicarboxylate symporter	AWC34_RS07770	AVJ22_RS08050	SE1039_RS08190	SEQMU2_RS13320	SEQU_RS26225	UF72_RS13410
Sodium:proton antiporter	AWC34_RS02375	AVJ22_RS02165	SE1039_RS02335	SEQMU2_RS07120	SEQU_RS22870	UF72_RS00840
Sodium:proton antiporter	AWC34_RS03560	AVJ22_RS03360	SE1039_RS03525	SEQMU2_RS08750	SEQU_RS24880	UF72_RS02055
Sodium:proton antiporter	AWC34_RS03865	AVJ22_RS03660	SE1039_RS03825	SEQMU2_RS09055	SEQU_RS22135	UF72_RS02360
Sodium:proton antiporter	AWC34_RS09570	AVJ22_RS09975	SE1039_RS09995	SEQMU2_RS01855	SEQU_RS14260	UF72_RS09025
Sodium:proton antiporter	AWC34_RS09730	AVJ22_RS10135	SE1039_RS10155	SEQMU2_RS02015	SEQU_RS14420	UF72_RS09185
Sodium:proton antiporter	AWC34_RS09825	AVJ22_RS10230	SE1039_RS10250	SEQMU2_RS02110	SEQU_RS14515	UF72_RS09280
Sodium:proton antiporter	AWC34_RS10590	AVJ22_RS10985	SE1039_RS11015	SEQMU2_RS02895	SEQU_RS15285	UF72_RS10050
Sodium:proton antiporter	AWC34_RS10840	AVJ22_RS11215	SE1039_RS11250	SEQMU2_RS03125	SEQU_RS15530	UF72_RS10295
Sodium ABC transporter ATP-binding protein	AWC34_RS08310	AVJ22_RS08605	SE1039_RS08730	SEQMU2_RS00620	SEQU_RS26170	UF72_RS13035
Sodium transporter	AWC34_RS09700	AVJ22_RS10110	SE1039_RS10130	SEQMU2_RS01990	SEQU_RS14395	UF72_RS09160
Sodium ABC transporter permease	AWC34_RS09800	AVJ22_RS10205	SE1039_RS10225	SEQMU2_RS02085	SEQU_RS14490	UF72_RS09255
Sodium:glutamate symporter	AWC34_RS09830	AVJ22_RS10235	SE1039_RS10255	SEQMU2_RS02115	SEQU_RS14520	UF72_RS09285
Proline:sodium symporter	AWC34_RS10580	AVJ22_RS10975	SE1039_RS11005	SEQMU2_RS02885	SEQU_RS15275	UF72_RS10040

Figure S1. Genomic alignment of six *S. equorum* strains.

MAUVE alignment of the genome sequences of *S. equorum* strains KM1031, C2014, KS1039, Mu2, UMC-CNS-924 and G8HB1. Boxes with the same colour represent homologous regions of sequences, without rearrangement (locally collinear blocks), shared among *S. equorum* genomes.

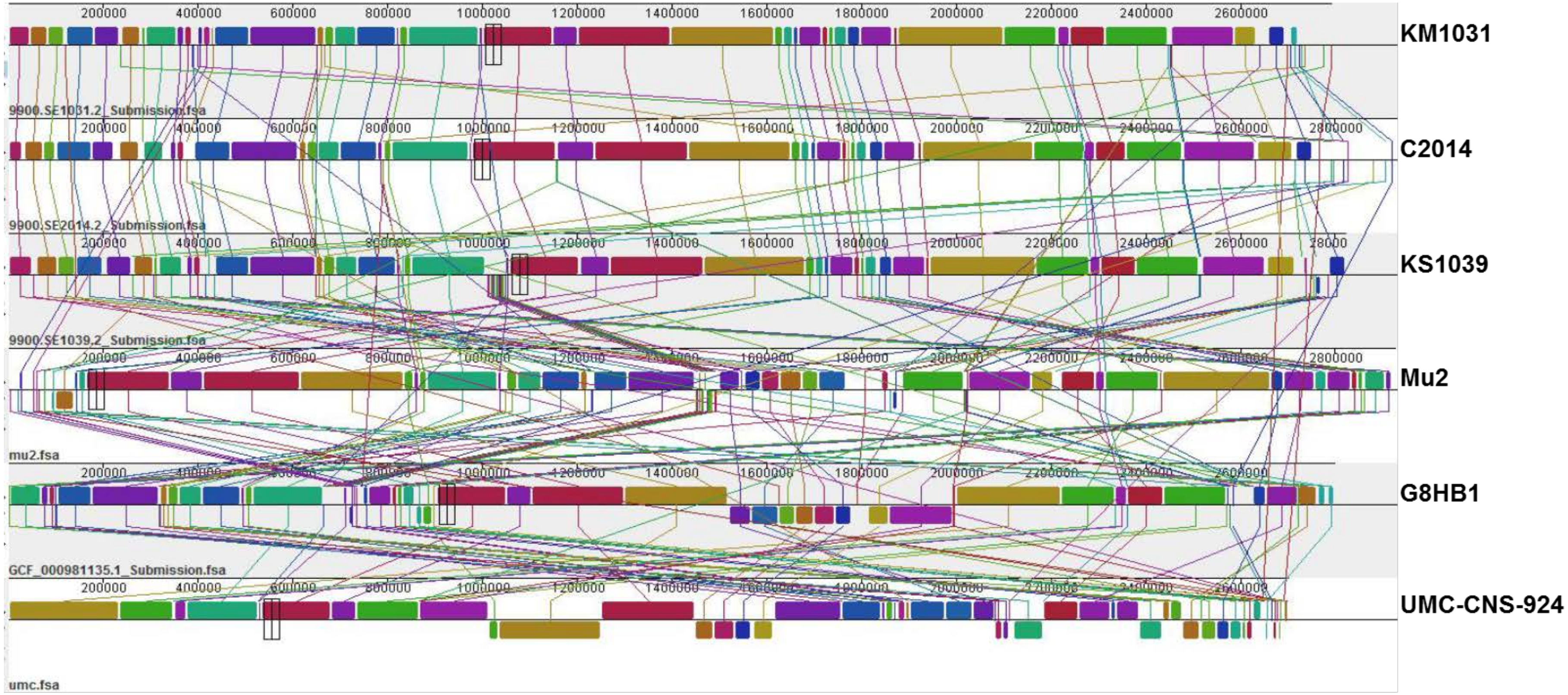

Figure S2. β-Haemolytic (A) and δ-haemolytic (B) activities of *S. equorum* strains on sheep blood-supplemented TSA plates. *Staphylococcus aureus* USA300-P23 was used as a positive control.

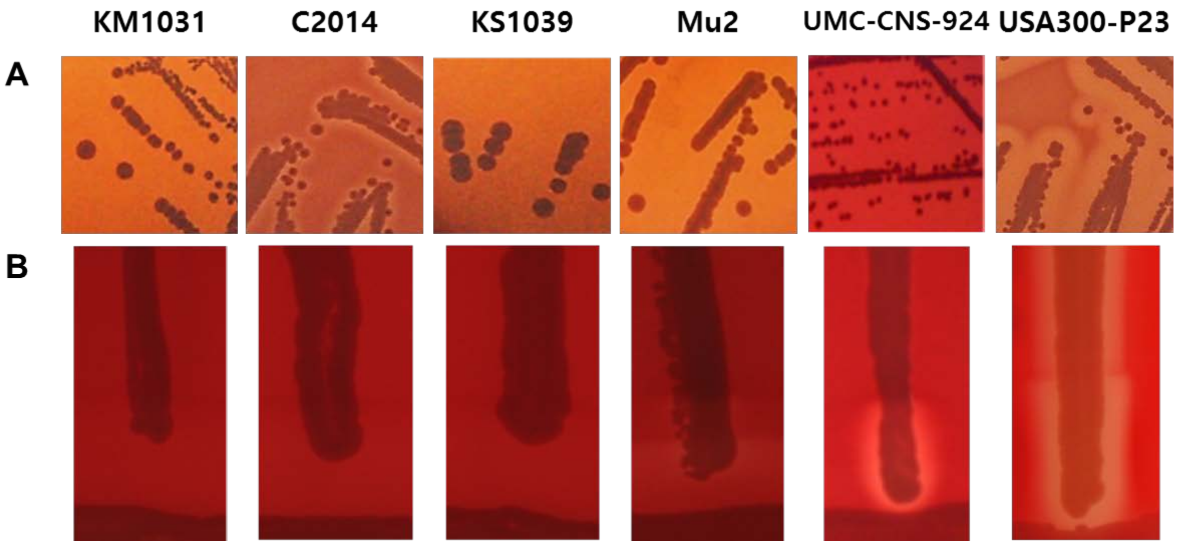

Figure S3. Growth of *S. equorum* strains in the presence of NaCl.

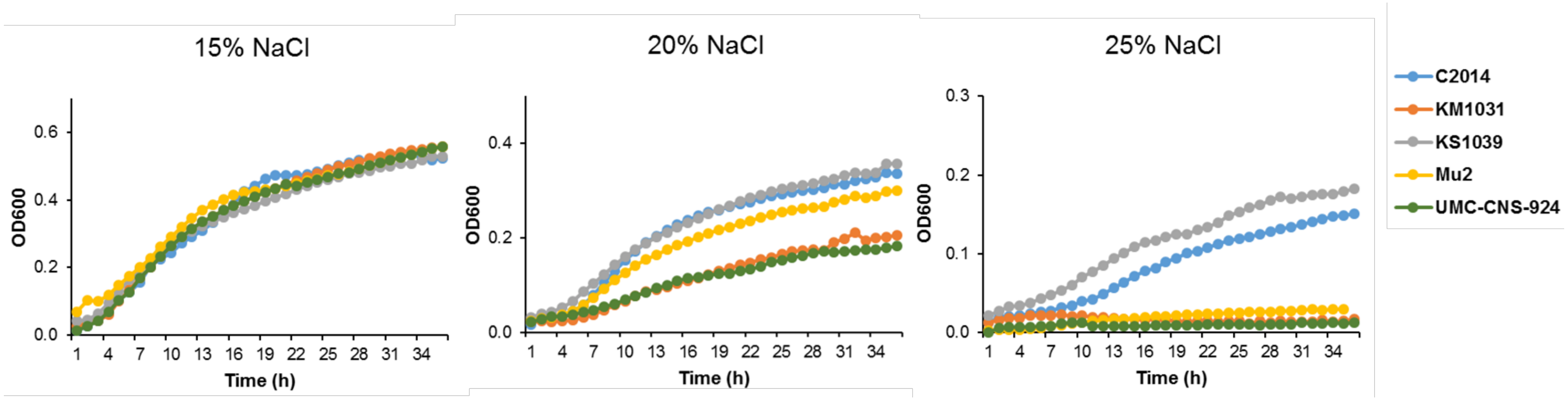

Figure S4. Genetic structures of the potassium voltage-gated channel genes. HPs presented by grey arrows indicate hypothetical proteins. Ball, ball domain; TM, transmembrane; L, re-entrant loop.

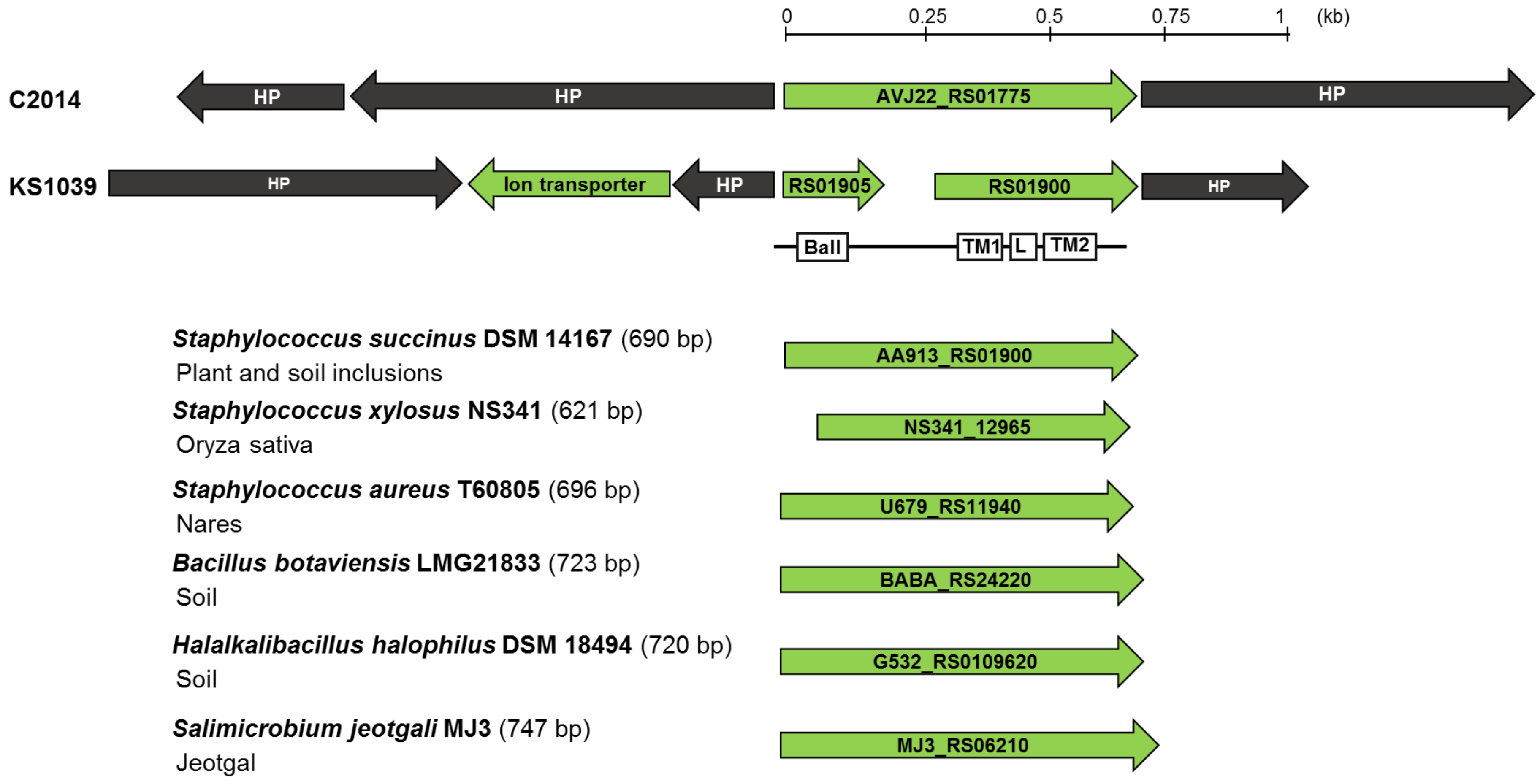

Supplement: Supplementary file 1 — Supplementary Information [file 41598_2017_5918_MOESM1_ESM.pdf]
